# Biomimetic and Locally Active Drug Delivery Systems for the Oral Biofilm and Periodontal Pocket: Formulation Strategies, Mechanisms and Translational Perspectives

**DOI:** 10.3390/pharmaceutics18081011

**Published:** 2026-08-16

**Authors:** Caterina Nela Dumitru, Alina Oana Dumitru, Teodora Marcu, Kamel Earar, Nicoleta Madalina Matei, Olimpia Dumitriu Buzia

**Affiliations:** 1 Research Centre in the Medical-Pharmaceutical Field, Faculty of Medicine and Pharmacy, “Dunărea de Jos” University of Galați, 35 Al. I. Cuza Street, Galați County, 800010 Galați, Romania; caterina.dumitru@ugal.ro (C.N.D.);; 2 Faculty of Medicine and Pharmacy, “Dunărea de Jos” University of Galați, 35 Al. I. Cuza Street, Galați County, 800010 Galați, Romania

**Keywords:** local drug delivery, oral biofilm, periodontal pocket, mucoadhesion, stimuli-responsive systems, biomimetic carriers, hybrid nano-in-gel systems, phytocompounds, antimicrobial resistance

## Abstract

Periodontitis and dental caries remain among the most prevalent chronic diseases, and their local treatment is limited less by the choice of active agent than by the difficulty of sustaining therapeutic concentrations against salivary and crevicular clearance, the mucosal barrier and the biofilm matrix. This narrative review (PubMed/MEDLINE, Scopus, Web of Science; 2010–2026; 118 sources) maps five mechanistic classes—mucoadhesive, in situ gelling, stimuli-responsive, nano-/microparticulate and biomimetic—alongside marketed sustained-release products onto the specific barrier each addresses and onto an explicit translational gradient. The barriers are quantified rather than described: a pocket of ≈0.5 µL perfused at ≈20 µL/h turns over some 40 times hourly, giving an intra-crevicular half-life of about one minute, and the inflamed pocket is neutral-to-alkaline (pH 7.4–8.5), so acid-triggered release is a cariogenic and not a periodontal strategy, whereas alkaline-triggered release remains an open design space. A dose calculation from these figures identifies payload potency and deliverable mass, not carrier retention, as the binding constraint on phytocompound delivery. Because no single class overcomes all barriers, hybrid nano-in-macro architectures are analysed as the structural solution. Measured against a marketed benchmark of ≈0.3 mm additional probing-depth reduction, progress now depends on consolidation rather than on novelty.

## 1. Introduction

Periodontal disease and dental caries remain among the most common chronic conditions worldwide; severe forms of periodontitis affect a substantial proportion of the adult population and represent a major cause of tooth loss [1]. Beyond their local impact, these diseases are increasingly recognised as sources of low-grade systemic inflammation, with epidemiological associations to cardiovascular, cardiometabolic, and neurodegenerative diseases [2,3].

The central aetiological factor of these conditions is the dysbiotic oral biofilm, a structured microbial community embedded in an extracellular matrix that adheres to the hard and soft surfaces of the oral cavity [4]. The extracellular polymeric substance (EPS) matrix acts as a physical and chemical barrier that limits the penetration of antimicrobial agents and confers on the biofilm a markedly increased tolerance relative to the planktonic forms of the same microorganisms [5,6].

Standard treatment of periodontitis is based on mechanical debridement of the root surface and of the subgingival environment, a procedure conventionally designated scaling and root planing (SRP), sometimes supplemented with systemic antibiotic therapy [7]. SRP is the reference comparator against which every locally delivered adjunct discussed here has been tested, and the abbreviation is used in this sense throughout. Systemic antibiotic therapy, however, has important limitations: reaching effective concentrations in the periodontal pocket requires high systemic doses, while site bioavailability often remains subtherapeutic owing to salivary flow, clearance by gingival crevicular fluid, and limited diffusion within the biofilm [8]. Moreover, repeated systemic exposure contributes to selection pressure for antimicrobial resistance, a major public-health concern [7].

These limitations have redirected attention toward local drug delivery systems, designed to release the active agent directly into the oral cavity or periodontal pocket, achieving therapeutic concentrations at the site with minimal systemic exposure [8]. In recent years, these approaches have evolved from simple sustained-release systems to biomimetic and stimuli-responsive systems that mimic biological structures or respond to the specific features of the pathological environment (local pH shifts, bacterial enzymatic activity, oxidative stress) [9].

In parallel, there is growing interest in incorporating bioactive phytocompounds (polyphenols, essential oils, alkaloids such as berberine) into such systems, both for their antimicrobial and anti-inflammatory properties and as a strategy to overcome their limitations of solubility, stability, and local bioavailability [10,11,12]. Local delivery of these compounds may also reduce dependence on antibiotics and the associated selection pressure for resistance [10].

Despite the rapidly growing literature on individual delivery systems, an integrative framework that systematically links formulation strategy, mechanism of action on the biofilm, and translational maturity within a single coherent scheme is still lacking [8]. This gap persists even as the field has become densely populated: recent reviews have surveyed local drug-delivery systems and adjunctive pharmacotherapeutics for periodontitis broadly [13,14,15,16], nanoparticle-based delivery specifically [17], and, most recently, biomimetic cell-membrane–cloaked nanocarriers for the periodontal application [18]. Each of these, however, is organised primarily as an enumeration of system types or is confined to a single mechanistic class; none maps the full spectrum of formulation strategies onto the specific oral barrier each is designed to overcome, nor positions them along an explicit translational gradient. Against this backdrop, the present review is distinguished not by the introduction of any single system class, several of which are, individually, already well reviewed, but by five features that, taken together, constitute its contribution: (i) an explicit barrier-to-solution mapping in which every formulation class is paired with the specific oral barrier (salivary and crevicular clearance, mucus, pH variation, biofilm matrix) it primarily addresses; (ii) the placement of genuinely biomimetic carriers (cell-membrane-cloaked particles, extracellular vesicles, and extracellular-matrix- and antimicrobial-peptide–mimetic systems) within this same unified scheme rather than in isolation, allowing them to be compared on equal terms with established products; (iii) the treatment of multi-target phytocompounds as a cross-cutting, antimicrobial-resistance–sparing payload strategy woven through every system class rather than as a stand-alone topic; (iv) a dedicated analysis of hybrid, combination architectures, which follows directly from the observation that no single class resolves all oral barriers at once (Section 3.7); and (v) an explicit innovation-versus-clinical-validation gradient that positions each class along its translational maturity, reframing the central question from which mechanism is newest to which is closest to the patient. The review is structured accordingly: the anatomo-physiological challenges of the oral cavity (Section 3.1), the five system classes (Section 3.2, Section 3.3, Section 3.4 and Section 3.5), phytocompound payloads (Section 3.6), hybrid architectures (Section 3.7), and finally the translational status and the research gaps that condition it (Section 4).

## 2. Materials and Methods

### 2.1. Study Design

This work is a narrative review reported with structured transparency. It is neither a systematic review nor a meta-analysis: no protocol was pre-registered, no risk-of-bias tool was applied, and no quantitative pooling was attempted. The PRISMA-2020 checklist was used solely as a reporting aid, to make the search and selection process auditable, and does not imply systematic review methodology. The narrative format was chosen deliberately, because the objective of the review is to map heterogeneous formulation classes onto the oral barriers they address and onto a translational gradient, a comparative-conceptual task for which the outcome heterogeneity of the underlying literature (in vitro biofilm assays, animal models and clinical endpoints, with no shared endpoint set) precludes meaningful quantitative synthesis (Section 3.8.5).

### 2.2. Information Sources and Search Strategy

Three electronic databases were interrogated: PubMed/MEDLINE, Scopus and Web of Science Core Collection. The search covered records published between 1 January 2010 and 18 July 2026, the date on which it was last executed. Reference lists of all included articles and of the recent reviews identified [13,14,15,16,17,18] were hand-searched for additional sources (backward citation tracking).

The search combined three concept blocks with the Boolean operator AND, the terms within each block being combined with OR: Block A, delivery context—local drug delivery, periodontal pocket, intrapocket, oral biofilm, dental biofilm, dental plaque, buccal delivery; Block B, formulation class—mucoadhesive, in situ gel*, thermosensitive gel, stimuli-responsive, ROS-responsive, enzyme-responsive, pH-responsive, nanoparticle*, liposome*, nanofib*, microsphere*, biomimetic carrier, cell membrane-coated, extracellular vesicle*, exosome*, antimicrobial peptide*; and Block C, therapeutic payload—phytocompound*, phytochemical*, polyphenol*, flavonoid*, essential oil*, curcumin, berberine, epigallocatechin, quercetin, chlorhexidine, minocycline, doxycycline. The full strings as executed in each database are given in Supplementary Material S1.

Searches of Block A AND Block B were run to capture formulation studies; Block A AND Block C to capture payload studies; and Block A AND Block B AND Block C to capture the intersection that constitutes the core evidence of Section 3.6 and Section 3.7. Filters applied at the database level were the publication date, English language, and (for PubMed) the exclusion of the publication types “Comment”, “Editorial” and “Letter”.

Two features of this strategy require explicit justification. First, the 1 January 2010 start date captures the modern formulation literature but excludes the pivotal registration trials of the marketed intra-pocket products that anchor the “established” tier of this review. Those trials were therefore retrieved deliberately, outside the date filter, by backward citation tracking and by targeted searching on product and molecule names, and the clinical claims of Section 4.1.1 are supported by the trials themselves [19,20,21] rather than by secondary summaries of them. Second, the English-language restriction was applied for reasons of screening capacity within the review team. Because Brazilian, Chinese and Japanese groups publish extensively in dental materials and periodontology, this is a substantive rather than a formal limitation, and it is restated as such in Section 2.6.

### 2.3. Eligibility Criteria

Records were eligible if they satisfied all of the following: (i) they described a defined delivery system, that is, a formulation with a specified carrier, composition and release behaviour rather than a free drug or extract applied without a vehicle, or established the epidemiological, microbiological, pathophysiological or methodological context required to interpret such systems; (ii) the intended or tested application site was the oral cavity, the periodontal pocket, the oral mucosa or the dental hard tissues, or the study reported the mechanistic principle underlying such an application; and (iii) they reported original experimental data (in vitro, ex vivo, in vivo or clinical) or an authoritative mechanistic account of formulation behaviour.

Records were excluded if they were not in English; if no delivery system was defined and the record supplied no contextual or transferable mechanistic principle; if the application site was unrelated to the oral cavity and no transferable principle was contributed; if they were conference abstracts, theses, preprints or records without retrievable full text; or if they were duplicate reports of the same dataset, in which case the most complete publication was retained.

A specific decision rule was adopted for citations drawn from outside the oral field: general-mechanism references (for example, on the chemistry of ROS-cleavable linkers or the physical chemistry of ionotropic gelation) were retained only where they establish a principle that has no equivalent primary description in the oral literature, and in every such case at least one study performed in an oral or periodontal model is cited alongside them. All citations were checked against this rule.

### 2.4. Selection Process and Records Retrieved

**Figure 1:**
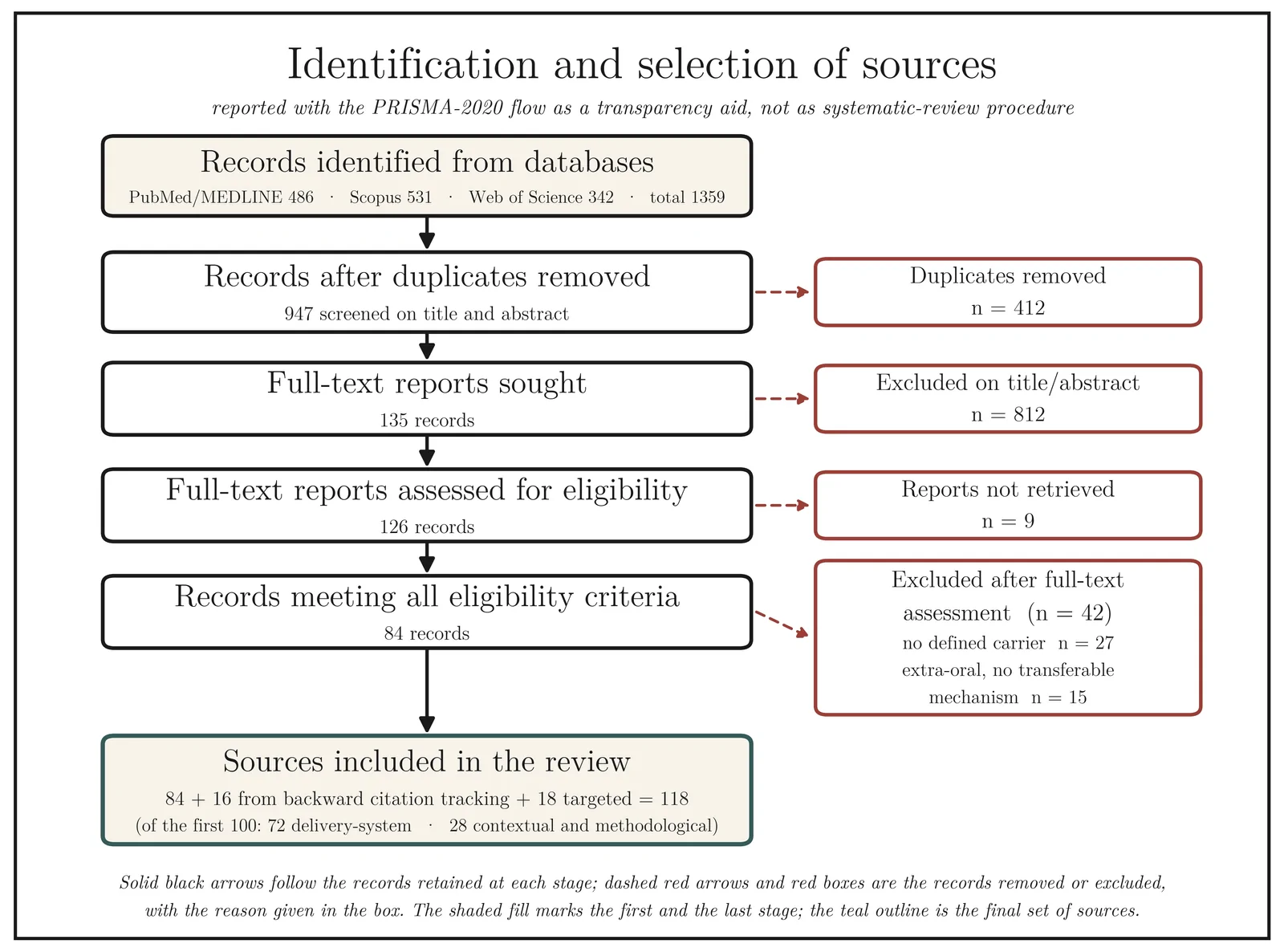
Identification and selection of sources. Flow of records through the identification, screening, retrieval and eligibility stages described in this section, together with the reasons for exclusion at each stage. The PRISMA-2020 flow is used as a transparency aid only, in the sense set out in Section 2.1. The figure is original and was created by the authors for this review; no previously published material is reproduced in it and no permission is required.

The flow of records through the selection process is summarised in Figure 1. The database searches returned 1359 records (PubMed/MEDLINE 486; Scopus 531; Web of Science 342), of which 412 were duplicates. The remaining 947 were screened at the title and abstract level by two authors independently (C.N.D. and A.O.D.), with disagreements resolved by discussion with a third author (T.M.); 812 were excluded at that stage, leaving 135 records for full-text retrieval; 9 could not be obtained, and 42 of the 126 assessed were excluded, for absence of a defined carrier (*n* = 27) or an extra-oral setting without mechanistic relevance (*n* = 15). This left 84 records that satisfied all eligibility criteria. Backward citation tracking of the included articles and of the recent reviews identified in Section 2.2 added 16 sources, and 18 further sources were retrieved by directed searching on specific quantitative parameters and on the competing adjuncts discussed in Section 4.2.4, bringing the total to 118. Of the 100 sources retrieved by database searching and backward citation tracking, 72 describe a defined delivery system and constitute the formulation evidence base of Section 3 and Section 4, while the remaining 28 supply, under criterion (i) of Section 2.3, the epidemiological, microbiological, phytochemical and methodological context in which those systems are interpreted. The next paragraph classifies the full set of 118 by study design rather than by subject matter.

Because a narrative synthesis can only be as quantitative as its sources, the composition of the evidence base is stated explicitly. Of the 118 sources cited, 43 report original experimental or clinical data (in vitro, ex vivo, in vivo or clinical), of which six are controlled or randomised clinical trials; a further seven are systematic reviews with meta-analyses or evidence-based clinical practice guidelines; and the remaining 68 are narrative reviews or authoritative mechanistic accounts, used for context and for principles that have no primary description in the oral literature. We accept that this proportion is high for a review of formulation behaviour. Because secondary sources rarely carry the numbers required by the barrier characterisation of Section 3.1 and the dose calculation of Section 3.1.7, every quantitative statement in those two sections is anchored to a source that measured the quantity, and the three instances in which no such measurement exists (the effective pore size of intact dental biofilm, the residence of the oral mucin film, and the survival of a membrane coating in the periodontal pocket) are identified as gaps rather than filled with estimates.

### 2.5. Data Extraction and Synthesis

**Figure 2:**
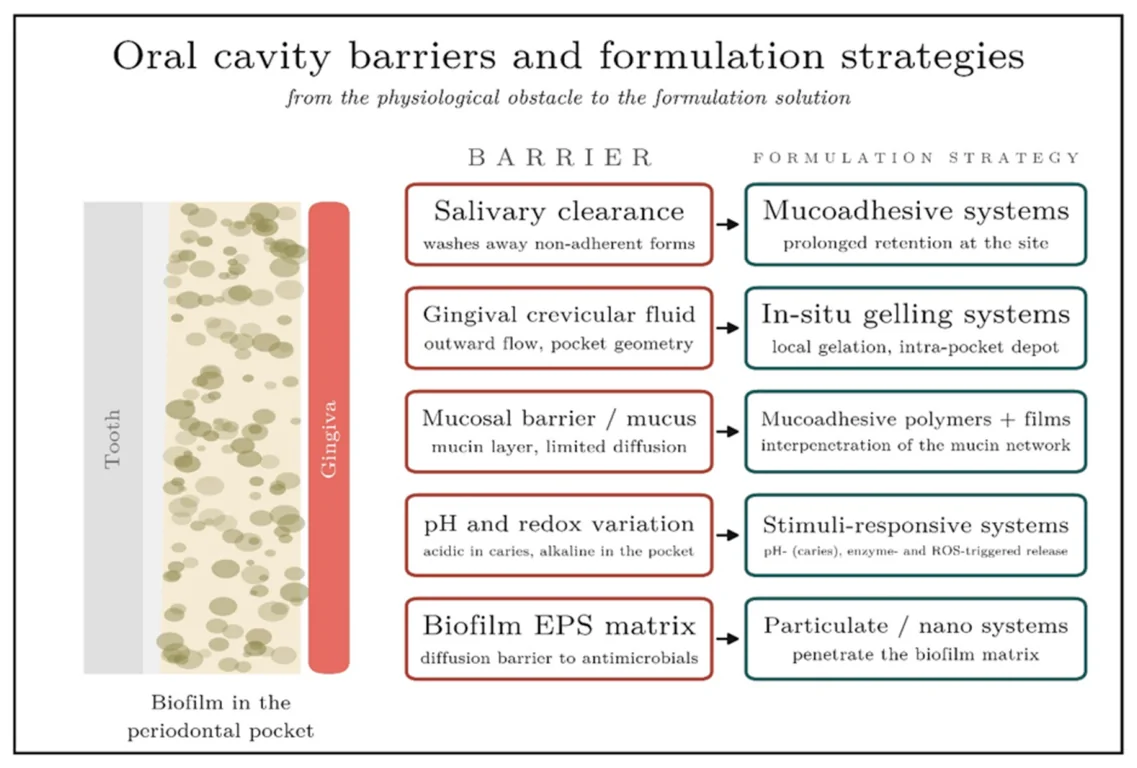
Schematic of the oral cavity as a delivery site. Each physiological barrier (salivary clearance, gingival crevicular fluid, mucus, pH variation, biofilm matrix) is paired with the formulation strategy designed to overcome it, illustrating the barrier-to-solution logic that structures Section 3.2, Section 3.3, Section 3.4 and Section 3.5. The quantitative values of each barrier, and the sources from which they are taken, are given in Table 1; the figure should be read together with that table rather than as a stand-alone summary of the section headings. The figure is original and was created by the authors for this review; no previously published material is reproduced in it and no permission is required.

**Figure 3:**
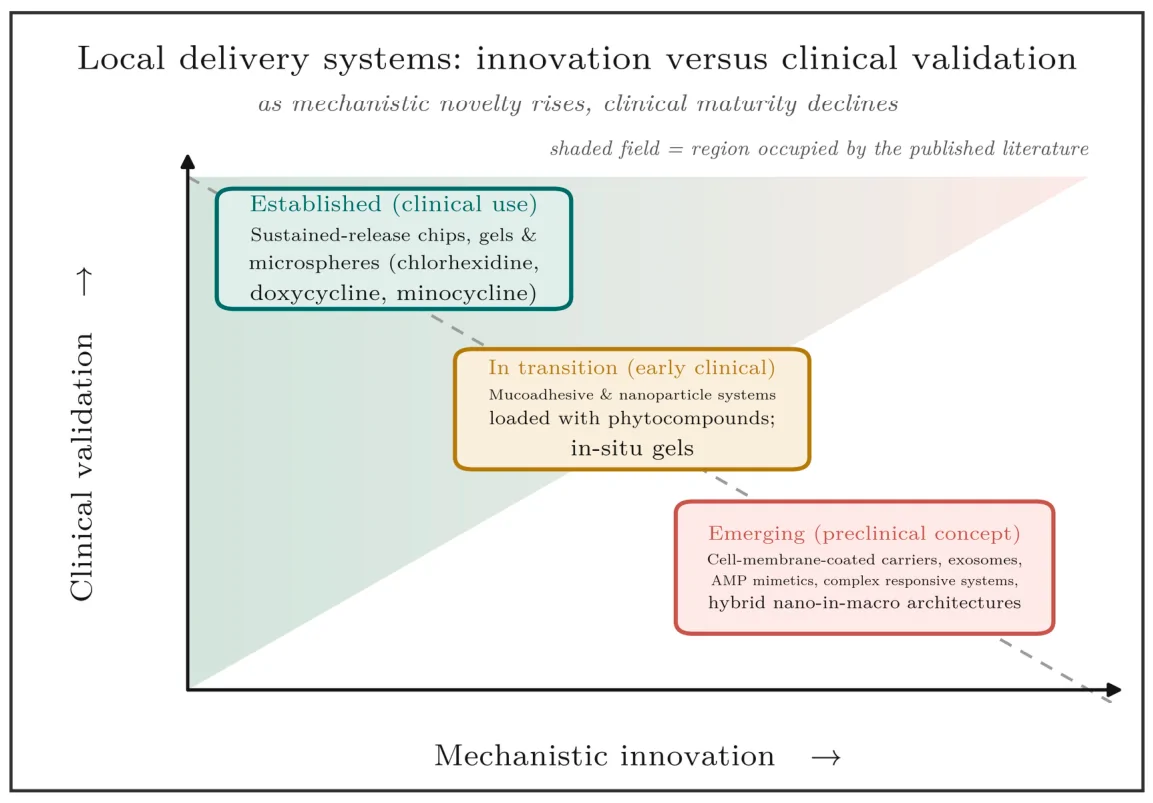
Taxonomy of local delivery systems positioned on the innovation-versus-clinical-validation gradient. Established sustained-release products sit at high clinical maturity; mucoadhesive and nanoparticle phytocompound systems occupy the transition zone; cell-membrane carriers, exosomes, AMP mimetics, and complex responsive systems remain at the preclinical frontier. The gradient encodes translational maturity (Section 4.1.5).The colours and arrows carry meaning and are read as follows. Each box is coloured by tier: teal for systems in established clinical use, amber for those in transition at the early-clinical stage, and red for emerging preclinical concepts. The arrows along the two axes indicate the direction of increase only and do not carry a scale. The dashed diagonal represents the inverse relationship between mechanistic innovation and clinical maturity asserted in the text, and is an interpretative guide rather than a fitted line. The two axes are defined operationally as follows, so that the position of each class can be checked and, if necessary, disputed. The vertical axis is clinical validation, scored on the ordinal scale used throughout Section 4.1: (1) in vitro evidence only; (2) evidence in an animal model of periodontitis; (3) at least one controlled clinical study in patients; (4) at least one adequately powered randomised controlled trial; (5) regulatory approval and marketing for the periodontal indication. The horizontal axis is mechanistic innovation, scored on the degree of departure from diffusion-controlled release of a conventional antimicrobial: (1) passive diffusion from an inert matrix; (2) retention or gelation engineered through carrier physicochemistry; (3) release triggered by a pathological signal; (4) a carrier possessing an intrinsic therapeutic function; (5) a carrier presenting a biological identity or mimicking a biological structure. Each class is plotted at the highest level attained by any published member, not at the level of its median member, which is why single studies can move a class upward; the shaded band marks the region occupied by the published literature and is not a fitted relationship. Hybrid nano-in-macro architectures (Section 3.7) occupy the same preclinical band as their responsive and nanoparticulate components. The figure is original and was created by the authors for this review; no previously published material is reproduced in it, and no permission is required.

For each included source the following were extracted into a structured matrix: system class; carrier composition; payload; the oral barrier primarily addressed; release mechanism and kinetics; the experimental model used (in vitro monospecies or multispecies biofilm, ex vivo substrate, animal model, clinical trial); the principal quantitative outcome; and the level of evidence attained. This matrix underlies the barrier-to-solution mapping of Figure 2, the translational gradient of Figure 3 and the comparative syntheses presented in Section 3. Priority in the narrative was given to studies conducted in an oral or periodontal model; where a class is supported only by extra-oral evidence, this is stated explicitly in the text.

### 2.6. Limitations of the Search

Three limitations follow from the design. The English-language restriction (Section 2.2) may have excluded relevant work, particularly from East Asian dental-materials groups. The absence of formal risk-of-bias appraisal means that studies are described but not graded, so in vitro proof-of-concept and randomised clinical evidence must be weighed differently, as the translational gradient of Section 4.1 makes explicit. Finally, because selection prioritised systems with a defined carrier and a reported mechanism, formulations that failed and were reported only as negative results are almost certainly under-represented, a publication bias that the review can flag but cannot correct.

## 3. Local and Biomimetic Delivery Systems for the Oral Cavity

This section synthesises the retrieved literature. It first characterises the oral cavity as a delivery site, whose measured parameters are consolidated in Table 1, and then examines each formulation class in turn, together with phytocompound loading, the hybrid architectures that combine these classes and the experimental models used for their evaluation, mapping each class to the oral barrier it primarily addresses and to its translational maturity.

The organising scheme requires three clarifications before it is applied, since the framework rather than any single system class is the contribution claimed here. (i) Cardinality: Five mechanistic classes are distinguished throughout—mucoadhesive, in situ gelling, stimuli-responsive, nano-/microparticulate and biomimetic—to which are added, as a sixth and seventh entry in Table 2, the hybrid architectures of Section 3.7 and the established sustained-release products of Section 4.1.1, which are marketed dosage forms rather than distinct mechanisms. In situ gelling and stimuli-responsive systems share a single Section 3.3. because they share the sol–gel formalism, but they are separate classes and are separated in Table 2 and in Figure 3; the numbering of the subsections is therefore a matter of exposition and does not imply a four-class taxonomy. (ii) Division of labour between the tables: Table 2 classifies by mechanism and reports advantages, limitations and maturity; Table 3 classifies the same material by dosage form and reports the formulation determinants, the release kinetics and the scalability of each manufacturing route. The two tables answer different questions and are deliberately not merged, but no claim appears in both. (iii) Orthogonality: The classes are orthogonal in mechanism, not in material. Chitosan appears under mucoadhesives, under polymeric nanoparticles and within hybrid systems because a single polymer can be deployed to defeat different barriers; each appearance is a different mechanism and is cross-referenced accordingly. For the same reason, the entry “phytocompound-loaded systems” in the final row of Table 3 is a payload strategy and not a system class, is placed last for that reason, and is excluded from the clinical-validation ordering of the other rows, as the caption states. A trigger that is present at all times (temperature, salivary ions) is likewise distinguished throughout from a trigger that reports active disease (enzymes, reactive oxygen species): both are handled in Section 3.3 for reasons of formulation chemistry, but only the second constitutes pathological targeting, and Table 2 separates them.

### 3.1. Anatomo-Physiological Challenges of the Oral Cavity as a Delivery Site

The oral cavity is a particularly difficult environment for drug delivery and retention, and understanding its physiological barriers is essential for the rational design of any locally active system. These barriers account for most of the formulation decisions discussed in the following sections. Each barrier is characterised below by its measured parameters rather than by adjectives, since every design decision described in Section 3.2, Section 3.3, Section 3.4, Section 3.5, Section 3.6 and Section 3.7 is downstream of those numbers; the parameters are consolidated with their sources in Table 1, and the consequence for dosing is worked through explicitly in Section 3.1.7.

#### 3.1.1. Salivary Flow and Clearance

Saliva provides continuous washing of oral surfaces, with basal and stimulated flow rates that rapidly remove non-adherent dosage forms and dilute the active agent [22,23]. This clearance severely limits the contact time of conventional formulations and constitutes the principal argument for mucoadhesive and in situ gelling systems [24].

The relevant magnitudes are as follows. Unstimulated whole-salivary flow is of the order of 0.3–0.4 mL/min, rising to 1.5–2.0 mL/min under masticatory or gustatory stimulation, and a residual film of approximately 0.8 mL remains in the mouth after swallowing [22]. A freely dissolved, non-adherent agent is therefore diluted into a compartment that is replaced several times per minute, and its oral clearance half-time is of the order of a few minutes; retention beyond that horizon has to be engineered, not assumed. This single figure is the quantitative justification for the entire mucoadhesive and in situ gelling literature reviewed in Section 3.2 and Section 3.3.

#### 3.1.2. Gingival Crevicular Fluid and the Periodontal Pocket

Within the periodontal pocket, gingival crevicular fluid (GCF) flows from the tissue toward the lumen, at a rate that increases in proportion to inflammation [23]. This flow exerts a washout effect opposite to the diffusion of the drug from the lumen toward the base of the pocket, reducing the concentrations achieved at the site and requiring systems with prolonged intra-pocket retention [23]. The narrow, deep geometry of the pocket additionally raises challenges of physical access for viscous or particulate formulations [8].

Quantitatively, the periodontal pocket is a very small and very rapidly flushed reservoir, and this is the central arithmetic fact of local periodontal delivery. The resting volume of a moderate 5 mm pocket is approximately 0.5 µL, with reported values across pocket sizes of 0.4–1.5 µL; mean crevicular fluid volume recovered per site rises from about 0.49 µL in health to about 0.73 µL in disease. Flow into the pocket averages approximately 20 µL/h and increases with inflammation, so that the pocket contents turn over of the order of 40 times per hour and the half-life of an unbound, freely soluble agent within the crevice is approximately one minute [25]. Sustaining a therapeutic concentration for days against a clearance of this magnitude is not a matter of degree but of kind, and it is what a subgingival reservoir exists to do; Section 3.1.7 converts these figures into the release rate and payload mass such a reservoir must provide, and compares that requirement with the marketed products.

#### 3.1.3. The Mucosal Barrier and Mucus

The oral mucosa, covered by a mucin-rich mucus layer, constitutes both an adhesion surface exploitable through interactions with mucoadhesive polymers and a diffusion barrier for active molecules [26]. Turnover of the mucus layer imposes a natural limit on the duration of adhesion of mucoadhesive systems [24,26].

That limit can be given an approximate magnitude. Turnover of the oral epithelium itself is of the order of 5–14 days depending on the region, being fastest in the non-keratinised junctional and sulcular epithelium and slowest in keratinised masticatory mucosa [27]; the residence of the adherent mucin film is considerably shorter and, unlike gastrointestinal mucus, has not been directly measured in the human mouth. The practical ceiling on mucoadhesive residence in the oral cavity is therefore set empirically, by detachment and washout studies, at hours rather than days [26], and we state it as an empirical rather than a derived quantity. Any claim of multi-day mucosal retention should be supported by direct in vivo residence data rather than by in vitro detachment force alone.

#### 3.1.4. pH and Environmental Variation

The pH of the oral cavity varies regionally and temporally, influenced by saliva, diet, bacterial metabolic activity within the biofilm, and the inflammatory milieu [28]. A distinction must be drawn here that is often blurred and that has direct formulation consequences. In the cariogenic biofilm the direction of change is acidic: fermentation of dietary carbohydrate by acidogenic streptococci and lactobacilli drives plaque pH down to approximately 4.5–5.5 within minutes of a sugar challenge, with recovery to resting values over 20–60 min, and microelectrode mapping shows that these low-pH zones are steep and highly localised [28]. The inflamed periodontal pocket behaves in the opposite direction. Its microbiota is asaccharolytic and proteolytic, and the ammonia and amines released from the catabolism of crevicular fluid proteins alkalinise the environment [29]: gingival crevicular fluid from inflamed sites has been reported at pH 7.5–8.7 and 7.96 ± 0.10 when measured at physiological carbon dioxide tension [30], with values commonly quoted for the diseased pocket in the range 7.4–8.5 and a tendency to rise with probing depth and disease activity [31]. The literature is not unanimous: direct measurement with glass microelectrodes gave a mean of 6.92 ± 0.03 across health and disease and found no association with pocket depth [32], and localised acidic micro-niches may persist within the subgingival biofilm. What no series reports, however, is a periodontal pocket that is acidic overall; the range across the literature runs from neutral to markedly alkaline. Two design consequences follow, and both are developed in Section 3.3.3. A carrier engineered to release below pH 5.5 will remain inert in exactly the lesion this review is concerned with, so acid-triggered release is a caries strategy and not a periodontal one. Conversely, alkaline-triggered release, keyed to the pH range actually observed in the diseased pocket, is a rational and, at present, an essentially unexplored design space. The chemical lability of certain payloads across this pH range remains a formulation concern and is treated in Section 3.6.1 [33].

#### 3.1.5. The Biofilm as a Diffusion Barrier

Beyond the host barriers, the biofilm itself represents a major barrier: the extracellular polymeric substance matrix slows or blocks the penetration of antimicrobial agents, while nutrient and oxygen gradients generate subpopulations with reduced metabolism that are tolerant to treatment [5,6,34]. Overcoming this barrier, through nanocarriers that penetrate the matrix or agents that disrupt it, is one of the central objectives of the systems discussed below [34].

The barrier can be given partial quantitative form, and its limits should be stated honestly. The matrix behaves as a size-selective and charge-selective sieve whose transport pathways are tortuous water channels between dense exopolysaccharide-rich microcolonies; its bulk composition, architecture and charge distribution in dental plaque are well characterised [35], but the effective pore size of an intact, intraorally grown dental biofilm has not been directly measured, and published estimates derive from tracer diffusion in model biofilms rather than from the oral community. The experimentally consistent finding is more restrictive than the usual formulation shorthand suggests: gold nanoparticles of 20 nm applied to biofilms grown intraorally on enamel adsorb strongly to the outer surface and to the pellicle and remain detectable after repeated washing, yet do not diffuse into the deeper strata [36]. Penetration is therefore governed jointly by steric hindrance, by electrostatic binding to the anionic matrix and by matrix heterogeneity, and small size alone does not confer it. The “optimal size window” invoked in Section 3.4.1 is consequently an empirical range obtained from penetration studies and not a value derived from a measured mesh size, and we designate it as such throughout.

#### 3.1.6. Implications for Delivery-System Design

In summary, an effective locally active system must simultaneously satisfy several requirements: sufficient retention against salivary and crevicular clearance, capacity to adhere to the mucosa or to hard surfaces, penetration of the biofilm matrix, and, ideally, release triggered by the specific signals of the pathological environment. These frequently conflicting requirements underlie the formulation trade-offs analysed in Section 3.2, Section 3.3, Section 3.4 and Section 3.5 and motivate the combination strategies analysed in Section 3.7.

**Table 1:** Quantitative parameters of the oral barriers to local delivery. The table gives, for each barrier identified in Section 3.1, the measured parameter that constrains formulation design, its value with the source, and the design consequence that follows. Values are representative adult figures; where a parameter has not been directly measured in the oral cavity this is stated rather than estimated.

Barrier	Parameter	Value (Source)	Direct Design Consequence
Salivary clearance (Section 3.1.1)	Unstimulated/stimulated whole-salivary flow; residual oral volume	0.3–0.4 mL/min unstimulated; 1.5–2.0 mL/min stimulated; ≈0.8 mL residual film after swallowing [22]	Oral clearance half-time of a non-adherent form is of the order of minutes; retention must be engineered (mucoadhesion, in situ gelation).
Crevicular clearance (Section 3.1.2)	Pocket resting volume; gingival crevicular fluid flow; turnover; intra-crevicular half-life	≈0.5 µL for a 5 mm pocket (range 0.4–1.5 µL); ≈0.49 µL in health vs. ≈0.73 µL in disease; ≈20 µL/h, rising with inflammation; ≈40 pocket volumes/h; t½ ≈1 min [25]	A subgingival reservoir is obligatory; the required zero-order release rate is set by MIC × flow (Section 3.1.7).
Mucus and mucosa (Section 3.1.3)	Epithelial turnover; residence of the adherent mucin film	Epithelial turnover ≈ 5–14 days by region [27]; mucin-film residence not directly measured in the human mouth; empirical mucoadhesive residence hours rather than days [26]	Mucoadhesion buys hours, not days; multi-day claims require in vivo residence data, not detachment force alone.
pH—cariogenic biofilm (Section 3.1.4)	Plaque pH after a carbohydrate challenge	Fall to ≈4.5–5.5 within minutes; recovery over 20–60 min; steep local gradients [28]	Acid-triggered release is valid here, and only here.
pH—inflamed periodontal pocket (Section 3.1.4)	Gingival crevicular fluid pH in disease	7.5–8.7 reported; 7.96 ± 0.10 at physiological PCO_2_ [30]; commonly quoted 7.4–8.5, rising with probing depth [29,31]; one microelectrode series reports 6.92 ± 0.03 with no depth association [32]	Neutral-to-alkaline, never acidic overall: acid-triggered carriers are inert here; alkaline-triggered release is an open design space.
Biofilm matrix (Section 3.1.5)	Effective pore size; penetration of model nanoparticles	Not directly measured in intact dental biofilm; matrix anionic and heterogeneous [35]; 20 nm particles adsorb to the surface without penetrating deeper strata [36]	Size alone does not confer penetration; the “size window” is empirical, and charge must be moderated (Section 3.4.1).
Payload potency (Section 3.1.7)	MIC against the target organism	Minocycline vs. periodontal pathogens ≈ 4 µg/mL [37]; berberine nanoparticles vs. *S. mutans* 78.1 µg/mL [38]; curcumin–silica vs. *P. gingivalis* 6.25–50 µg/mL [39]; clove-oil nanoemulsion vs. *S. mutans* 0.78 mg/mL [40]	Determines the deliverable mass over the intended duration and, for mg/mL payloads, becomes the binding constraint.

#### 3.1.7. What the Pocket Arithmetic Requires of a Formulation: A Worked Example

The parameters of Table 1 are not decorative; taken together they determine what an intra-pocket system has to do, and the calculation appears not to have been set out explicitly in the recent review literature. It is given here in its simplest defensible form.

Treat the pocket as a well-mixed compartment of volume V ≈ 0.5 µL perfused at Q ≈ 20 µL/h = 0.020 mL/h, and let a depot release drug at a constant rate R. At steady state the concentration in the pocket is C = R/Q, so the release rate required to hold the site at a target concentration C is simply R = C × Q, and the mass required over a treatment period t is M = C × Q × t. For a seven-day application, Q × t = 0.020 mL/h × 168 h = 3.36 mL, so M (in µg) ≈ 3.4 × C (in µg/mL). The arithmetic is deliberately conservative in one direction and optimistic in another: it ignores binding of the drug to the pocket wall, to the root surface and to the biofilm, which prolongs residence, and it ignores the fact that the biofilm itself is a sink in which the effective concentration is lower than in the fluid phase.

Applied to the marketed products, the calculation reproduces their design. To hold a periodontal pocket at 4 µg/mL of minocycline [37] for seven days requires R ≈ 0.08 µg/h and a total of ≈13 µg; the marketed microsphere product delivers 1 mg (1000 µg) per site [21], a margin of roughly 75-fold that accounts for the sustained supra-MIC crevicular concentrations reported for it and for its tolerance of an imperfect release profile. To hold the site at 125 µg/mL of chlorhexidine, a concentration chosen to exceed the elevated minimum inhibitory concentrations of biofilm-resident oral species, requires R ≈ 2.5 µg/h and ≈420 µg over seven days; the chlorhexidine chip carries 2.5 mg [19], a margin of about sixfold, consistent with its labelled seven- to ten-day release and with its narrower therapeutic window. The two marketed products are therefore not arbitrary: their payloads are the smallest that satisfy this inequality with a realistic safety factor.

Applied to the phytocompound systems tabulated in Table 4, the same calculation is less comfortable and, in our view, is the most useful single result in this review. Berberine nanoparticles with an MIC of 78.1 µg/mL against *S. mutans* [38] require ≈260 µg over seven days, and curcumin-loaded silica is active at 6.25–50 µg/mL against *P. gingivalis* [39] requires 20–170 µg: both are comfortably within what a subgingival insert can carry. A clove essential oil nanoemulsion with an MIC of 0.78 mg/mL [40], by contrast, requires ≈2.6 mg of active for the same period, which approaches or exceeds the payload of the largest marketed intra-pocket device and must be accommodated within a carrier that itself has to fit a pocket of sub-microlitre resting volume. The conclusion is specific and testable: for phytocompounds whose minimum inhibitory concentrations lie in the mg/mL range, the binding constraint on local delivery is payload potency and deliverable mass, not carrier retention, and no improvement in mucoadhesion or gelation will relieve it. Such compounds must either be potentiated (by nanoformulation, by synergistic combination or by efflux-pump inhibition; Section 3.6.5), be applied repeatedly rather than as a single depot, or be repositioned as antibiofilm and anti-virulence agents assessed against matrix and gene-expression endpoints rather than as bactericides assessed against MIC. We recommend that any future report of an intra-pocket phytocompound system state its MIC, its total payload and its release rate, so that this inequality can be checked by the reader.

### 3.2. Mucoadhesive Systems

Mucoadhesive systems aim to prolong the contact time between the dosage form and the oral surfaces, counteracting the salivary clearance described in Section 3.1.1. By fixing the formulation to the mucosa, they transform a transient exposure of a few minutes into sustained contact lasting hours, permitting graded release of the active agent at local therapeutic concentrations with minimal systemic exposure.

#### 3.2.1. Mechanisms of Mucoadhesion

Mucoadhesion is not a unitary phenomenon but the sequential result of several physicochemical steps. The first is the contact phase, in which the polymer wets and spreads over the mucosal surface; this step depends on the surface energy and swelling capacity of the polymer, which hydrates its chains and exposes reactive groups [26]. This is followed by the consolidation phase, in which the hydrated polymer chains physically interpenetrate the mucin network, creating an interdiffusion zone that mechanically anchors the formulation [26]. This anchoring is stabilised by secondary interactions (hydrogen bonds, electrostatic forces and hydrophobic interactions) [26]. In the case of cationic polymers, electrostatic attraction toward the sialic acid residues and sulphate groups of mucins adds a major adhesion component, which is why polymer charge is an essential design parameter [24,26].

#### 3.2.2. Chitosan and Its Derivatives

Chitosan, a cationic polysaccharide obtained by deacetylation of chitin, is among the most studied oral mucoadhesive polymers, combining strong adhesion, biodegradability, and intrinsic antimicrobial activity. Its mucoadhesion derives directly from the density of protonated amino groups at slightly acidic pH, which electrostatically binds anionic mucins [41]. Its antibacterial activity arises from the same positive charge: the protonated groups interact with the anionic components of the bacterial wall and membrane, destabilising the cell envelope, increasing permeability, and leading to leakage of intracellular contents [42]. This dual function makes it particularly valuable in biofilm-mediated diseases [42]. Its principal limitation, reduced solubility at neutral pH, is overcome through derivatives: thiolated chitosan forms disulphide bridges with mucins, while quaternised forms remain positively charged and soluble independently of pH [43]. The oral relevance of this dual function is documented directly: chitosan nanocapsules loaded with trans-cinnamaldehyde reduced *Streptococcus mutans* biofilm formation, down-regulated quorum-sensing and glucosyltransferase-associated virulence genes, and reduced caries scores in a rat model with low toxicity [44], while chitosan nanoparticles carrying epigallocatechin-3-gallate (EGCG) disassembled the *S. mutans* biofilm matrix in vitro, though without reducing bacterial viability or acidogenesis [45] (Table 4, note iv).

#### 3.2.3. Other Mucoadhesive Polymers

Hyaluronic acid, an anionic glycosaminoglycan present in periodontal tissues, adheres through hydrogen bonding and interpenetration but additionally provides properties of tissue-healing modulation and attenuation of inflammation [46]. Polyacrylic acid and the carbomers are strong mucoadhesives through the high density of carboxyl groups that form hydrogen-bond networks with mucins [47]. Cellulose derivatives and alginate modulate viscosity, adhesion, and the release profile, often in polymeric combinations [47,48]. Carbopol 934P is in fact the mucoadhesive component of one of the few intra-pocket phytocompound formulations taken as far as clinical assessment, a curcumin in situ gel described in Section 3.3.6 [49].

#### 3.2.4. Mucoadhesive Dosage Forms

Thin films and patches offer prolonged contact, precise dosing and, in multilayer configurations, unidirectional release toward the mucosa with a protective face that limits salivary washout, a configuration suited to localised lesions such as aphthous ulcers [50]. Mucoadhesive gels, tablets, microspheres, and fibres allow localised application, increased retention, and insertion into the periodontal pocket [47]. The choice of dosage form also determines the release kinetics, through control of the exchange surface area and the rate of hydration [47]. The clinically established chlorhexidine chip, a mucoadhesive cross-linked gelatin matrix inserted into the pocket, is the marketed embodiment of this principle and is discussed with its trial data in Section 4.1.1 [19].

#### 3.2.5. Applications in Biofilm-Mediated Diseases and Phytocompound Loading

Mucoadhesive systems have been loaded with a variety of agents, from classical antiseptics to phytocompounds such as polyphenols, essential oils, and berberine. Interest in phytocompounds is doubly motivated: their intrinsic antimicrobial and anti-inflammatory activity, but also the fact that their very pharmaceutical limitations—low solubility, instability, reduced local bioavailability—are precisely those that a mucoadhesive matrix can correct [10,11]. By immobilising the compound in a hydrated polymeric network, the system prolongs contact and isolates the molecule from degradation factors, releasing it gradually [44,51].

Concrete oral examples define what this achieves in practice. Berberine formulated as nanoparticles of 40–65 nm by wet milling showed a minimum inhibitory concentration (MIC) and minimum bactericidal concentration (MBC) against *S. mutans* of 78.1 and 312.5 µg/mL respectively, inhibited biofilm formation, and produced visible cell-surface damage on field-emission scanning electron microscopy, with improved dispersibility relative to the free alkaloid [38]. A clove (*Eugenia caryophyllus*) essential-oil nanoemulsion of 117 nm reached an MIC of 0.78 mg/mL and an MBC of 1.56 mg/mL against *S. mutans* and removed approximately 68% of pre-formed biofilm biomass at 3.12 mg/mL on bovine incisor substrate, without genotoxicity or cytotoxicity in the accompanying assays [40]. The determinants that emerge across these studies are consistent: the mucoadhesive matrix supplies residence time and protection from oxidation, the encapsulated phytocompound supplies the antimicrobial and antibiofilm activity, and the reported outcomes are consequently a joint property of carrier and payload rather than of either alone. A fuller tabulation of phytocompound systems with their carriers, models and quantitative outcomes is given in Table 4.

#### 3.2.6. Advantages and Limitations

The advantages are prolonged retention, localised dosing, minimal systemic exposure and, for bioactive polymers, a therapeutic contribution of the carrier itself. The limitations follow from mechanism: the duration of adhesion is finite, bounded by mucus turnover (Section 3.1.3), and performance varies inter-individually with salivary flow [24,26]. In addition, surface adhesion does not guarantee penetration of the mature biofilm, which is why mucoadhesives are frequently combined with particulate systems capable of penetrating the biofilm [34], the combination logic developed systematically in Section 3.7.

### 3.3. In Situ Gelling and Stimuli-Responsive Systems

In situ gelling systems reconcile a fundamental tension: the narrow geometry of the pocket calls for a fluid formulation, whereas retention calls for a viscous one. They are instilled as a low-viscosity liquid and then transition to a structured gel in response to a local stimulus. The generation responsive to pathological stimuli adds release triggered selectively by the signals of active disease. Two kinds of stimulus are grouped in this section for reasons of formulation chemistry, and they should not be conflated. Temperature and salivary ions are physiological and permanently present: a poloxamer gel that sets on warming has solved the problem of placement, not of targeting, and it will set equally well in a healthy sulcus. Enzymes, reactive oxygen species and, in principle, an alkaline shift are markers of active disease: a carrier keyed to them releases where and when the lesion is active. Only the second group constitutes pathological targeting, Table 2 assigns the two groups to different rows and different barriers accordingly, and the distinction matters to the formulator because it determines whether the trigger threshold has to discriminate between tissues or merely between the syringe and the mouth.

#### 3.3.1. The Sol–Gel Transition Principle

The sol–gel transition is the passage of a polymeric system from a fluid dispersion to a three-dimensional network that immobilises the liquid phase, through physical mechanisms (hydrophobic aggregation, micelle packing, ionic cross-linking) or chemical ones [52]. The advantage is the decoupling of the moment of application from the final state: the dose is instilled precisely as a liquid but behaves as a solid-viscous depot at the site [53]. The critical parameters are the stimulus threshold, the transition rate, and the mechanical strength of the gel [54].

#### 3.3.2. Temperature-Sensitive Systems

Poloxamers (Pluronic) are liquid at room temperature and gel at body temperature through temperature-dependent micellisation: on warming, the poly(oxypropylene) blocks dehydrate and associate hydrophobically, forming micelles that pack into a cubic network, the gel [52]. For periodontal applications, the solution instilled at room temperature gels on contact with the tissue [53]. Its modest mechanical strength is corrected by combination with mucoadhesive polymers, illustrating the synergy between Section 3.2 and Section 3.3 [54]. In the periodontal pocket specifically, a poloxamer 407 (Pluronic F127)/Carbopol 934P curcumin gel with a viscosity of 19,000–37,000 cP at 4 °C was clinically effective as an adjunct to mechanical therapy (Section 3.3.6) [49]; a hyaluronic acid/Pluronic F127 thermo-responsive gel carrying chitosan nanoparticles eradicated *S. mutans, Pseudomonas aeruginosa* and *Porphyromonas gingivalis* biofilms over 36 h in vitro and disrupted EPS formation [55].

#### 3.3.3. pH-Sensitive Systems

pH-responsive systems contain ionisable polymers that change their charge state, conformation, and solubility as a function of acidity: carboxyl groups are compact at low pH and expand at higher pH, and amino groups the reverse [9]. In the oral cavity, the principle couples to the pH gradients generated by the cariogenic biofilm and by inflamed sites [28]. Following the correction made in Section 3.1.4, this principle has to be split by disease. In dental caries a system can legitimately be designed to remain inert at neutral pH and to release within the acidic micro-environment of the lesion, where plaque pH falls to approximately 4.5–5.5 after a carbohydrate challenge; the threshold is then conventionally placed between pH 5.5 and 6.0 and the target is the acidogenic biofilm [33]. In periodontitis the same design fails, because the inflamed pocket is neutral-to-alkaline (pH 7.4–8.5 at inflamed sites, with values rising toward the deeper end of that range as probing depth and disease activity increase) rather than acidic [29,30,31]. An acid-triggered carrier placed subgingivally would therefore remain closed in precisely the environment it was intended to sense, and the pH-responsive periodontal systems reported to date largely avoid the difficulty by relying on a different trigger, most often enzymatic or oxidative (Section 3.3.5). The corollary is a genuine and, to our knowledge, essentially unexploited opportunity: polymers that ionise and swell above neutrality, for example, networks bearing tertiary amine or phenolic groups with apparent pKa values in the range 7.5–8.5, or borate and imine linkages whose hydrolysis accelerates under mild alkalinity, would be keyed to a signal that is actually present in the diseased pocket, that correlates with disease activity and that is absent from healthy sites. We identify alkaline-triggered intra-pocket release as a specific design target rather than as a general aspiration (Section 5), and note that its feasibility depends on demonstrating discrimination across a pH difference of well under one unit, which is a demanding but not unprecedented requirement [33].

The narrowness of that window is, on the periodontal side, the principal obstacle: the difference between a healthy sulcus and an active pocket may be a few tenths of a pH unit, superimposed on localised acidic micro-niches within the subgingival biofilm and on a fluid that is strongly bicarbonate-buffered above pH 8 [30]. Any alkaline-triggered system must therefore be characterised across the whole 6.5–8.5 interval and at physiological carbon dioxide tension, since pH values measured in the absence of CO_2_ are systematically too high by several tenths of a unit [30]. We recommend that this be treated as a reporting requirement for the class.

#### 3.3.4. Ion-Sensitive Systems

Ionotropic gelation exploits salivary cations: alginate gels in the presence of calcium through egg-box junctions between the guluronic blocks of adjacent chains [56]. Gellan gum follows a similar principle, suited to in situ oral formulations [57]. The advantage is that the stimulus is constantly present; the complementary disadvantage is the lack of specificity for the diseased site [58]. References [56,58] are retained for the physical chemistry of ionotropic gelation, which has no equivalent primary description in the dental literature; the oral application is documented by [57] and, for ionotropically gelled chitosan nanoparticles, by [45,55].

#### 3.3.5. Systems Responsive to Pathological Stimuli (Enzymes, ROS)

The most advanced class responds to molecular signals that mark active disease, a shift from spatial targeting to pathophysiological targeting. At inflamed sites, pathogens and host cells overexpress specific enzymes—hyaluronidases, bacterial proteases and matrix metalloproteinases (MMPs)—that can be exploited as keys which selectively cleave the carrier network and release the agent within the enzymatic focus. In parallel, inflamed tissue generates an excess of reactive oxygen species (ROS), which has led to materials bearing chemical bridges sensitive to oxidation (thioether, selenium, borate esters) that cleave in the presence of ROS in proportion to the local oxidative stress [59,60]. References [59,60] are retained solely for the chemistry of the responsive linkers; in line with the decision rule of Section 2.3, oral evidence is cited alongside them.

For the enzymatic trigger, matrix metalloproteinases are over-expressed in the inflamed periodontium, and MMP-2 in particular is present in the gingival crevicular fluid of periodontitis patients in a latent form that crevicular proteases and *P. gingivalis* gingipains activate locally [61], which makes it a rational key for intra-pocket release. Zhou and co-workers exploited exactly this: acrylated hyaluronic acid was cross-linked with an MMP-2-cleavable peptide to form a collagenase-responsive hydrogel loaded with nanoparticles of the PERK inhibitor GSK2606414; release was markedly accelerated under the enzymatic conditions of the periodontal microenvironment, the loaded gel improved the osteogenic differentiation of inflammation-exposed cells without affecting normal-cell proliferation, and it reduced inflammation and promoted alveolar bone regeneration in a rat periodontitis model [62].

For the oxidative trigger, two periodontal studies define the achievable behaviour. Zhao and colleagues built an injectable oxidised-dextran/phenylboronic-acid–functionalised poly(ethyleneimine) network whose B–N coordination both raised drug loading and conferred ROS-triggered release of doxycycline and metformin; the system adhered to gingival tissue, was active against *P. gingivalis*, *Staphylococcus aureus* and *Escherichia coli*, and improved alveolar bone morphometry, histology and immune-inflammatory mediator expression in a diabetic rat model of chronic periodontitis [63]. Zhu and colleagues grafted 3-aminophenylboronic acid onto hyaluronic acid and cross-linked it with poly(vinyl alcohol) through borate ester bonds, co-loading minocycline hydrochloride with ultrasmall Fe–quercetin nanoparticles; hydrogen peroxide progressively disintegrated the network and accelerated minocycline release, the construct adhered to porcine gingiva (≈9 kPa) and to enamel (≈8 kPa), scavenged 77.1% of DPPH radicals, shifted macrophage polarisation from M1 to M2 through the Nrf2/NF-κB axis, and reduced alveolar bone loss in a rat model [64].

The result is on-demand release, self-regulated according to disease activity, with sparing of healthy tissue. This class is particularly relevant to oxidative-stress-associated oral diseases, where the carrier can have a dual function—a triggered vehicle and a ROS-consuming element, restoring the local redox balance; the dual-function concept is defined formally in Section 4.2.5.

#### 3.3.6. Phytocompound Loading

Thermosensitive and ion-sensitive gels can incorporate plant extracts, polyphenols, or essential oils, releasing them in a sustained manner as the matrix erodes, compensating for the short local half-life [53,65]. Coupling antioxidant phytocompounds with ROS-responsive systems creates a coherent loop: the greater the oxidative stress, the more counteracting agent is released [66,67,68].

The following named systems, all evaluated in oral or periodontal models, substantiate this statement.

Curcumin. Nasra and co-workers formulated a 2% curcumin in situ gel by the cold method, screening temperature-sensitive (Poloxamer 188 and 407), pH-sensitive (Carbopol 934P) and ion-sensitive (gellan gum) polymers; polyethylene glycol 400 was selected as solubiliser (Section 3.6.2). The optimised formulation gelled between 28 and 34 °C, remained syringeable through a 21-gauge needle when cold, retained drug content, release profile and pH over three months of refrigerated storage, and, delivered into the periodontal pocket as an adjunct to mechanical therapy, produced a significant reduction in probing depth and bleeding index and a lesser reduction in plaque index [49].

*Opuntia ficus-indica* extract. Conte and colleagues encapsulated the extract in chitosan nanoparticles produced by microfluidic ionotropic gelation with tripolyphosphate, which protected the polyphenols from enzymatic oxidation, and dispersed these nanoparticles in a hyaluronic acid/Pluronic F127 thermo-responsive gel (OFI@tgel) that is liquid when cold and solid at body temperature. The construct eradicated *S. mutans*, *P. aeruginosa* (PAO1) and *P. gingivalis* biofilms over 36 h, disrupted extracellular polymeric substance formation, and shifted macrophage polarisation from the M1 to the M2 phenotype [55].

Quercetin. In the ROS-responsive hyaluronic acid–phenylboronic acid/poly(vinyl alcohol) hydrogel described above, quercetin was delivered as ultrasmall iron–quercetin coordination nanoparticles of approximately 2 nm, chosen precisely because the free flavonoid is poorly soluble and unstable. Within the gel the flavonoid supplied 77.1% (DPPH) and comparable (ABTS) radical-scavenging capacity, up-regulated SOD-1 and CAT, down-regulated IL-1β and TNF-α while raising IL-10 and Arg-1, protected human periodontal ligament stem cells from oxidative damage and restored their osteogenic differentiation under peroxide stress, and contributed to reduced alveolar bone resorption in vivo [64]. This is the clearest published realisation of the antioxidant-phytocompound/ROS-responsive-carrier loop asserted in the preceding paragraph: peroxide cleaves the borate ester, and the released polyphenol consumes the peroxide.

Comparative reading. Across these systems, the carrier is chosen against the specific pharmaceutical defect of the payload: solubilisation for curcumin, protection from oxidation for the *Opuntia* polyphenols, and metal coordination for quercetin, while the gel supplies intra-pocket residence. The reported endpoints differ in kind (clinical indices for curcumin, biofilm eradication and macrophage phenotype for *Opuntia*, redox and osteogenic endpoints for quercetin), which is itself an illustration of the endpoint heterogeneity discussed in Section 3.8.5 and one reason why the class cannot yet be ranked quantitatively.

#### 3.3.7. Advantages and Limitations

The advantages are minimally invasive application, prolonged retention, and specificity of release. The limitations are more difficult manufacturing reproducibility, dose control dependent on the fluctuating intensity of the biological stimulus, and still-limited clinical validation, most of the evidence being in vitro and preclinical [60]. Of the periodontal responsive systems cited above, none has progressed beyond the rodent model [62,63,64].

### 3.4. Nano- and Microparticulate Systems

Particulate systems address the barrier that mucoadhesives and gels do not overcome by themselves: penetration of the mature biofilm (Section 3.1.5). Through their reduced size, high surface-to-volume ratio, and surface functionalisation, nano- and microparticles traverse the matrix, accumulate within the biofilm, and release the agent at the bacterial target. They are frequently incorporated into mucoadhesive or gelling systems, combining macroscopic retention with nanometric penetration (Section 3.7).

#### 3.4.1. The Principle of Biofilm Penetration at the Particulate Scale

Penetration depends on size, surface charge, and hydrophilicity. Sufficiently small particles diffuse through the pores of the matrix, so an optimal size window exists [69]. Charge plays a dual role: cationic particles are attracted by the anionic matrix and cells, but an excessive charge may trap them at the periphery [69]. Functionalisation allows tuning of the interactions and the addition of targeting ligands [34]. In the oral setting the same size–charge logic governs performance: the cationic chitosan nanocarriers of Section 3.2.2 act on the *S. mutans* biofilm through the cationic charge described above, although only one of the two is within the size window discussed below, the epigallocatechin-3-gallate carrier having a reported diameter of approximately 500 nm and acting on the matrix rather than through deep penetration [44,45].

The window can be stated numerically, with the caveat entered in Section 3.1.5 that it is empirical rather than derived. Across the penetration studies surveyed here, hydrodynamic diameters below approximately 200 nm are required for entry into the matrix and diameters below about 100 nm are associated with the deepest reported penetration, while zeta potentials in the region of +10 to +30 mV give useful electrostatic attraction to the anionic matrix and to bacterial envelopes; above roughly +30 mV the same attraction becomes counterproductive and particles are retained at the biofilm periphery, and strongly anionic particles are repelled by the matrix altogether [35,69]. Two qualifications should accompany any use of these figures. Size and charge are not independent, since strongly cationic particles aggregate on contact with the polyanionic matrix and their effective diameter in situ exceeds their measured diameter in suspension; and, as noted above, 20 nm particles applied to intraorally grown biofilms adsorbed to the surface without penetrating [36], so the lower bound of the window is not simply “as small as possible”. Reported values should therefore be measured in the medium of use, and not in water.

#### 3.4.2. Polymeric Nanoparticles

Polymeric nanoparticles (PLGA, polycaprolactone, chitosan) encapsulate the agent within a solid matrix, releasing it through controlled diffusion and hydrolytic degradation [70]. By adjusting the composition of the polymer, the release profile is tuned from hours to weeks [71]. Chitosan nanoparticles combine encapsulation with mucoadhesion and antibacterial activity (Section 3.2.2) [42,44]. PLGA/PLA nanoparticles have also been the vehicle for the only randomised placebo-controlled trial of a nanoencapsulated phytocompound in the periodontal pocket, discussed critically in Section 4.1.2 [72].

#### 3.4.3. Lipid Nanoparticles

Liposomes, solid lipid nanoparticles (SLN), and nanostructured lipid carriers (NLC) are suited to lipophilic compounds, a category that includes many phytocompounds. Liposomes can encapsulate hydrophilic compounds in the core and lipophilic ones in the bilayer simultaneously, while their affinity for membranes favours fusion with the bacterial envelope [73]. SLN and NLC stabilise the compound within a solid lipid matrix, with superior physical stability [74]. For problematic phytocompounds, these systems are often the only way to bring them to active concentrations at the site [75]. In the oral setting, nanoliposomes of approximately 79 nm carrying a thymol- and carvacrol-rich *Zataria multiflora* extract showed antibacterial, antibiofilm and anti-attachment activity against *S. mutans* and inhibited attachment to enamel specimens comparably to 0.2% chlorhexidine [76].

#### 3.4.4. Metallic and Inorganic Nanoparticles

Silver nanoparticles release ions that bind to the thiol groups of bacterial proteins, disrupt the respiratory chain, and generate intracellular ROS [77]. ZnO and CuO nanoparticles act through similar mechanisms and have been explored as dental antibiofilm agents [78]. Their attractiveness, antibiotic-free action, is counterbalanced by concerns regarding biocompatibility, tissue accumulation, and toxicity [79]. Mesoporous and hollow mesoporous silica have been used in the same setting as inert reservoirs rather than as intrinsic antimicrobials: curcumin-loaded silica nanoparticles inhibited a clinical isolate of *P. gingivalis* at 6.25–50 µg/mL with release sustained to day 45 [39], and quercetin-loaded hollow mesoporous silica nanocomposites promoted dentinal tubule occlusion and preserved the demineralised organic matrix against erosion and abrasion while remaining biocompatible with gingival fibroblasts and pulp stem cells [80].

#### 3.4.5. Nanofibres and Fibrous Systems

Nanofibres obtained by electrospinning form meshes of high porosity in which the agent is incorporated directly into the fibre and released through degradation or diffusion [81]. The fibrous geometry is advantageous for membranes or intra-pocket inserts, serving as both a physical barrier and a reservoir, a concept close to guided tissue regeneration [82]. Electrospinning of polymer–phytocompound blends yields meshes with sustained release of natural compounds [83].

#### 3.4.6. Advantages and Limitations

The defining advantage is biofilm penetration and intra-matrix delivery, plus the versatility of encapsulation and targeting through functionalisation. The limitations are colloidal stability over time, the risk of aggregation, reproducible control at manufacturing scale [84] and, for metallic particles, long-term safety [79]. Maximum value is obtained through integration into retentive systems, realising combined nano–macro architectures (Section 3.7), the central theme of biomimetic systems.

### 3.5. Genuinely Biomimetic Systems

Here the term “biomimetic” is used in the narrow sense adopted throughout this review, that is, of systems that directly mimic biological structures, components or functions—cell membranes, natural vesicles, the architecture of the extracellular matrix, or innate defence mechanisms. These materials tend to be better tolerated, to evade clearance and, at times, to possess intrinsic therapeutic function. In the oral context, they represent the frontier of the field, with high potential and the largest preclinical-to-clinical gap. This is a definitional convention adopted for clarity within the present review, not a criticism of other usages: the word is applied in the wider literature to any bio-inspired or bio-derived material, and the narrower reading is used here only so that the systems in this section can be compared with one another on a consistent basis.

#### 3.5.1. Cell-Membrane-Cloaked Carriers

A nanoparticle core is wrapped in a natural cell membrane (erythrocyte, platelet, leukocyte, bacterial), which transfers to the carrier the surface identity of the source cell and allows it to evade immune recognition, prolonging residence at the site [85]. Erythrocyte-membrane-cloaked nanoparticles can act as nanodecoys that sequester pore-forming bacterial toxins [86]. The principle is attractive for periodontal disease, in which toxins and virulence factors contribute substantially to tissue destruction [2]. That statement should be made specific, because the choice of target determines whether a nanodecoy is the right tool. The dominant virulence factors of *Porphyromonas gingivalis* are the gingipain cysteine proteases and lipopolysaccharide rather than pore-forming toxins, and a membrane decoy is not the natural countermeasure against either. The organism for which the decoy principle is mechanistically exact is *Aggregatibacter actinomycetemcomitans*, whose leukotoxin A is a repeats-in-toxin family pore-former that binds the β_2_ integrin LFA-1 on leukocytes and kills them, is produced in greatest quantity by the strains most strongly associated with localised aggressive periodontitis, and requires cholesterol-containing membrane microdomains for cytotoxicity [87]. An erythrocyte- or leukocyte-membrane nanosponge presenting those same lipid domains is precisely the construct that would sequester it, and we identify leukotoxin A neutralisation as the specific and testable periodontal application of the nanodecoy concept, in preference to the general appeal to “toxins and virulence factors”. References [85,86] establish the platform and the nanosponge principle outside the oral cavity; the periodontal proof of concept follows. Tang and colleagues developed erythrocyte-mimicking nanovesicles that target *P. gingivalis* in periodontitis, exploiting the affinity of this pathogen for erythrocyte membranes to concentrate the carrier at the pathogen and combine decoy and delivery functions in a single construct [88]; a broader survey of bionic cell-membrane-mediated nanodrug delivery in periodontitis is available in [18].

One question that follows directly from the environment, and that the cell-membrane-cloaking literature has not addressed in the periodontal setting, should be stated plainly because it determines whether the platform can work here at all. Cloaking confers benefit because intact surface proteins on the donor membrane transfer a biological identity to the particle. The periodontal pocket, however, is a proteolytic reactor: *P. gingivalis* gingipains reach concentrations of the order of 1 µM in the pockets of patients, an order of magnitude or more above the concentrations required in vitro to degrade host membrane and matrix proteins [89], and they act alongside host matrix metalloproteinases and neutrophil elastase in a compartment that is continuously flushed by crevicular fluid. How long a cloaked particle retains its membrane identity under those conditions is unknown; no study identified here has measured the integrity of a membrane coating after exposure to gingipains or to pocket fluid, and the systemic circulation studies from which the platform derives [85,86] cannot answer the question because their environment is not proteolytic in the same way. We regard this as the central open question of the class. It is also readily tractable, requiring only that coating integrity and surface-protein retention be measured after incubation with purified gingipains or with pooled crevicular fluid, and we propose it as a minimum characterisation requirement for any cell-membrane-cloaked system proposed for intra-pocket use. Should the coating prove short-lived, the consequence is not that the approach fails but that its useful function is likely to be toxin sequestration over hours rather than immune evasion over days.

#### 3.5.2. Extracellular Vesicles and Exosomes

Exosomes are membrane-bound vesicles secreted by cells, carrying proteins, lipids, and nucleic acids, offering high biocompatibility and the capacity to traverse biological barriers [90]. Exosomes from mesenchymal stem cells modulate inflammation and stimulate tissue repair, adding a regenerative dimension, an effect confirmed by systematic review and meta-analysis of preclinical periodontal-regeneration studies [91]. Plant-derived vesicles are also being investigated as biocompatible and scalable carriers, a bridge toward the delivery of phytocompounds [92]. The limitations are the standardisation of isolation, the yield, and reproducible characterisation [90].

#### 3.5.3. Extracellular-Matrix-Inspired Systems

These materials reproduce the architecture and composition of the native matrix to create scaffolds that support regeneration, also serving as a reservoir of active agent [93]. Collagen or hyaluronic acid hydrogels combine biocompatibility with a carrier function, releasing agents as they are remodelled by cells [46,93]. Through their dual function, regeneration and delivery, they surpass the strictly antimicrobial paradigm [94]. Both anchoring references are set in the oral field: [93] addresses collagen-based biomaterials for periodontal regeneration, and [94] biomimetic mineralised scaffolds bearing antimicrobial peptides for dental application.

#### 3.5.4. Antimicrobial-Peptide Mimetics

Antimicrobial peptides (defensins, the cathelicidin LL-37) act through physical disruption of microbial membranes: being cationic and amphipathic, they attach to the anionic envelope and destabilise it, a mechanism against which resistance develops much more slowly [95]. Their instability to proteases and their cost have led to synthetic mimetics that reproduce the cationic-amphipathic character with superior stability [96]. Incorporation into delivery systems protects and concentrates them at the site; the amelogenin-derived peptide ADP-5, formulated as a hydrogel, was cytocompatible with periodontal cells, supported remineralisation and modulated the inflammatory profile in vitro [97]. The link to the anti-resistance strategy is direct: a physical mechanism, difficult to evade, and applied locally, reduces selection pressure (quantified, for the analogous multi-target phytocompounds, in Section 3.6.5).

#### 3.5.5. Advantages and Limitations

The advantages are high biocompatibility, intrinsic therapeutic functions, and mechanisms with a low risk of resistance. They are counterbalanced by the highest degree of complexity of any category, in reproducible manufacturing, standardisation, scalability and cost, and by the lowest level of clinical validation [90,98]. This gap sets the research agenda discussed in the final sections.

### 3.6. Phytocompound Loading: Pharmaceutical Challenges and Formulation Solutions

Bioactive phytocompounds—polyphenols, flavonoids, essential oils and alkaloids such as berberine—combine, within a single molecule, antimicrobial, anti-inflammatory, and antioxidant activity, matching the multifactorial nature of periodontal disease. The paradox is that precisely the compounds with the richest biological profile are often the most difficult to administer locally.

#### 3.6.1. Intrinsic Pharmaceutical Barriers

The first limitation is the low aqueous solubility of lipophilic compounds (curcumin being emblematic), which reduces the available fraction [12]. The second is chemical instability: many polyphenols oxidise on contact with oxygen, light, or alkaline pH [12,51], an instability documented in the oral setting by the enzymatic oxidation of *Opuntia* polyphenols that chitosan encapsulation was designed to prevent [55]. The third is reduced local bioavailability, the result of low solubility and stability plus rapid clearance and limited penetration [10,12]. Some compounds also have a bitter taste that affects compliance [99].

Reference [51], a food-science study of phenolic stability under simulated gastrointestinal conditions, is retained solely for the underlying chemistry of polyphenol oxidation and not as evidence about the oral cavity. The oral evidence is supplied by [55], in which chitosan encapsulation was introduced to prevent enzymatic oxidation of *Opuntia* polyphenols, and by [12,99] for curcumin, where both are cited together; the oral source carries the claim.

#### 3.6.2. Encapsulation as a Solution for Stabilisation and Solubilisation

Encapsulation increases apparent dispersibility in the aqueous medium and isolates the molecule from degradation [12]. Lipid nanoparticles are suited to lipophilic compounds, with the possibility of co-encapsulation [75]. Inclusion complexes with cyclodextrins increase solubility by hosting the lipophilic molecule within the hydrophobic cavity [99]. The magnitude of the problem, and of the solution, is measurable: curcumin’s solubility differs by more than an order of magnitude between candidate solubilisers (140 mg/mL in polyethylene glycol 400 versus 5.35 mg/mL in propylene glycol), and this single formulation decision determined whether an intra-pocket gel could be loaded at a therapeutically useful concentration [49]; quercetin required coordination to iron to become dispersible and stable enough to be carried in an aqueous hydrogel [64].

#### 3.6.3. Retention and Sustained Release

By incorporating the encapsulated phytocompound into a retentive system (Section 3.2 and Section 3.3), contact with the site is prolonged, and the release profile shifts from a rapid burst to a sustained one [44]. Such combined architectures, a nanocarrier that provides biofilm penetration and protection of the molecule, integrated into a retentive matrix that provides localisation, constitute the synthesis that this entire review argues for [34,75] and are analysed as a class in Section 3.7.

#### 3.6.4. Therapeutic Effect Aligned with Oral Pathophysiology

The antioxidant activity of polyphenols counteracts oxidative stress, the anti-inflammatory activity modulates the inflammatory cascade, and the antimicrobial activity acts on the pathogens—three targets addressed simultaneously by a single class of compounds [3,10,51,66,68]. This convergence makes local delivery of phytocompounds a means of restoring the redox and inflammatory balance of the oral environment.

#### 3.6.5. Contribution to the Antimicrobial-Resistance Strategy

Local delivery of phytocompounds reduces the need for systemic antibiotic therapy and the associated selection pressure [10]. Some phytocompounds act as adjuvants that restore antibiotic susceptibility, by inhibiting efflux pumps or disrupting the biofilm matrix, opening the possibility of synergistic combinations at reduced doses [68,100,101].

The available evidence permits a mechanistic and, in part, a numerical comparison, although it does not yet permit a head-to-head clinical one; both sides are stated here.

Mechanistically, the difference is one of target multiplicity. Conventional antibiotics used in periodontal therapy act on a single, well-defined molecular target: the tetracyclines (including minocycline and doxycycline) bind the 30S ribosomal subunit, so that a single point mutation in the target, a ribosomal protection protein or an efflux determinant confers resistance, and that determinant is horizontally transmissible. Most phytocompounds act instead on the bacterial envelope and on multiple cytoplasmic targets simultaneously: the lipophilic monoterpenes and phenolic terpenoids partition into the membrane and disrupt its integrity and fluidity, an essentially physicochemical mechanism for which there is no discrete resistance locus to mutate. The consequence, as with the antimicrobial peptides of Section 3.5.4, is that adaptation can occur, but as reversible phenotypic tolerance mediated by changes in membrane lipid composition or efflux, rather than as stably inherited resistance.

Quantitatively, the best-characterised comparison comes from serial-passage experiments. Hammer and colleagues passaged *S. aureus* and *E. coli* daily for six days in the presence of antibiotic alone, or of antibiotic plus sub-inhibitory tea tree essential oil or its principal monoterpene terpinen-4-ol. Median MICs for the antibiotics alone rose 4- to 16-fold over six days (4-fold for ciprofloxacin and vancomycin, 8-fold for mupirocin in *S. aureus*; 16-fold for ciprofloxacin and kanamycin and 8-fold for ampicillin in *E. coli*). Over the same six passages the MICs of the essential oil and of terpinen-4-ol themselves rose only 4-fold in *S. aureus* and 2-fold in *E. coli*, and in a separate macrodilution experiment resistance to terpinen-4-ol could not be induced at all: staphylococcal strains failed to grow above 0.1–0.2% terpinen-4-ol after 18–22 serial subcultures, and their susceptibility to ampicillin, ciprofloxacin, gentamicin, tetracycline and vancomycin was essentially unchanged, with the few 4-fold changes observed being in the direction of increased rather than decreased susceptibility [102]. The presence of the oil did not accelerate antibiotic-resistance development and, in one combination, reduced it: culture on kanamycin agar containing tea tree oil yielded approximately 10-fold fewer resistant *E. coli* mutants than kanamycin alone [102].

The comparator on the antibiotic side is available from the oral cavity itself. In a randomised clinical trial, patients receiving supra- and subgingival debridement with or without minocycline microspheres were sampled at baseline, one month and six months, and isolates were cultivated with and without 4 µg/mL minocycline. The proportion of minocycline-resistant isolates in saliva and subgingival plaque rose significantly at one month in the microsphere group and fell again by six months, while remaining unchanged in the control group; resistance among the recognised periodontal pathogens *Aggregatibacter actinomycetemcomitans, Tannerella forsythia* and *P. gingivalis* was absent or infrequent, but *Gemella morbillorum* and *Eubacterium saburreum* showed significant increases in both groups [37]. The clinically relevant reading is that even a locally delivered antibiotic, at doses far below systemic exposure, measurably selects for resistance in the oral community, albeit transiently.

Two caveats must be stated plainly. First, the serial-passage data derive from *S. aureus* and *E. coli* rather than from oral pathogens; equivalent passage experiments with *S. mutans* or *P. gingivalis* under phytocompound pressure have not, to our knowledge, been published, and this is a specific and tractable research gap (Section 4.2.1). Second, no clinical trial has compared resistance selection between a phytocompound-loaded and an antibiotic-loaded local delivery system in the periodontal pocket. The argument that phytocompound loading is resistance-sparing therefore rests on a mechanistic rationale supported by in vitro passage data and on the indirect benefit of reduced antibiotic exposure, not on direct clinical evidence, and we present it as such throughout this review.

A counter-argument runs in the opposite direction and is not resolved by the data above; it is set out here because it follows from this review’s own mechanistic analysis. A sustained-release depot that delivers an antimicrobial for days into a compartment it cannot fully penetrate generates, by construction, a prolonged concentration gradient across the target population, with supra-inhibitory concentrations at the biofilm surface and sub-inhibitory concentrations in its depths. Section 3.4.1 states the mechanism explicitly: excessive cationic charge traps particles at the periphery, and Section 3.1.5 records that even 20 nm particles may fail to reach the deeper strata. A prolonged sub-inhibitory exposure of a large, structurally protected bacterial population is the textbook condition for selecting reduced susceptibility, and local delivery therefore trades a reduction in systemic selection pressure for an increase in the duration of sub-inhibitory local exposure. The oral cavity supplies direct evidence that this is not hypothetical. Reduced susceptibility to chlorhexidine, the most widely used intra-pocket antiseptic, has been documented in oral species after repeated sub-inhibitory exposure, with associated changes in membrane composition and efflux and, in some studies, decreased susceptibility to unrelated antibiotics; the same review notes that the deep strata of dental plaque are exactly where sub-inhibitory biocide concentrations are expected to occur [103]. The minocycline-microsphere trial cited above makes the same point for a locally delivered antibiotic [37].

Three considerations nevertheless leave the balance in favour of local delivery, and we state them as reasons rather than as proof. First, the magnitude of exposure differs by orders of magnitude: a systemic course exposes the entire oral, gut and skin microbiota for the duration of therapy, whereas an intra-pocket depot exposes a defined site. Second, the selection observed after minocycline microspheres was transient, returning toward baseline by six months, and did not extend to the recognised periodontal pathogens [37]. Third, and most relevant to the argument of this review, the sub-inhibitory-gradient objection applies with full force to single-target antibiotics and with reduced force to multi-target membrane-active payloads, for which there is no discrete resistance locus for the gradient to select; this is the specific reason for preferring such payloads in a sustained-release format, and it is a mechanistic reason rather than an empirical one. The honest summary is therefore that local delivery reduces total selection pressure while creating a local condition favourable to it, that payload choice determines which effect dominates, and that the question is empirical and unresolved. We accordingly present the resistance-sparing claim as a hypothesis with a mechanistic rationale, and we specify in Section 4.2.1 the experiment that would test it: parallel serial-passage and ecological-endpoint studies of phytocompound-loaded and antibiotic-loaded intra-pocket systems in the same oral community.

In this way, the three threads are tied together: formulation technology, redox pathophysiology, and the anti-resistance strategy. A comparative synthesis of the delivery-system classes discussed above is provided in Table 2.

**Table 2:** Comparative overview of the delivery-system classes discussed in Section 3.2, Section 3.3, Section 3.4 and Section 3.5 and Section 4.1, mapping each class to its key mechanism, the oral barrier it primarily addresses, its main advantages and limitations, and its translational maturity. A reference column is included so that every claim regarding advantages, limitations and translational maturity is traceable to a cited source.

System Class	Key Mechanism	Barrier Addressed	Advantages	Limitations	Maturity	Refs
Mucoadhesive systems (chitosan, hyaluronic acid)	Polymer–mucin interpenetration + electrostatic/H-bond adhesion	Salivary clearance; short contact/residence time	Prolonged contact; localised dosing; bioactive carriers	Finite adhesion (mucus turnover); poor biofilm penetration	Established/clinical	[19,24,26,41,42,43,44,46,47,50]
In situ gelling systems (thermo-, pH-, ion-triggered)	Sol–gel transition; conforms to pocket geometry	Pocket geometry vs. retention trade-off	Minimally invasive; intra-pocket depot	Modest mechanical strength; formulation complexity	Early clinical	[49,52,53,54,55,56,57,58]
Stimuli-responsive systems (enzyme/ROS-cleavable)	Cleavable linkers degraded by disease-specific signals	Lack of pathological targeting	On-demand, self-regulated release; redox-active potential	Reproducibility; dose control; scarce clinical data	Preclinical	[9,59,60,61,62,63,64]
Particulate/nanosystems	Matrix diffusion + surface functionalisation for penetration/targeting	Biofilm matrix diffusion barrier	Biofilm penetration; versatile cargo	Colloidal stability; scale-up; metal-NP safety	Preclinical/early clinical	[34,39,69,70,71,72,73,74,75,77,78,79,80,84]
Biomimetic carriers (cell-membrane cloaking, exosomes, ECM- and AMP-mimetics)	Immune evasion; intrinsic virulence-factor sequestration; tissue function	Immune clearance; loss of low-resistance mechanisms	Immune evasion; intrinsic therapeutic function	Manufacturing; standardisation; least clinical validation	Emerging/preclinical	[18,85,86,88,90,91,92,93,94,95,96,97,98]
Hybrid nano-in-macro architectures	Nanocarrier for penetration embedded in a retentive gel, fibre or membrane	Two barriers simultaneously: clearance + biofilm/EPS matrix	Decoupled optimisation of retention and penetration; sequential release	Two-stage manufacture; characterisation of nanoparticle within matrix; no clinical data	Preclinical (rodent)	[55,62,64]
Sustained-release (classic)	Simple diffusion-controlled release of conventional antimicrobials	Subtherapeutic local levels	Proven clinical adjunct to SRP	Limited spectrum; antibiotic-resistance pressure; modest effect size	Established/clinical	[13,14,16,19,20,21,37]

Abbreviations: AMP, antimicrobial peptide; ECM, extracellular matrix; EPS, extracellular polymeric substance; NP, nanoparticle; ROS, reactive oxygen species; SRP, scaling and root planing (defined at first use in Section 1).

### 3.7. Combination and Hybrid Architectures: Pairing Classes Against Complementary Barriers

The central claim of this review, that no single class overcomes all oral barriers simultaneously, has a direct corollary: the classes must be combined, and the combinations follow a small number of recurring design patterns. This section makes those patterns explicit and examines the published intra-oral examples of each.

#### 3.7.1. Why Combination Is the Logical Consequence of the Barrier Analysis

Section 3.1 identified four largely independent barrier groups: clearance (salivary and crevicular), the mucus and mucosal barrier, environmental variation in pH and redox state, and the EPS matrix of the biofilm itself. Each formulation class addresses one or two of these well and the remainder poorly. Mucoadhesives and in situ gels solve residence but not penetration; nanoparticles solve penetration but are cleared within minutes if applied unbound; responsive chemistries solve the timing of release but do not by themselves place or retain the dose; and biomimetic coatings solve recognition and immune evasion but require a core with its own release behaviour. The combination is therefore not an incremental refinement but the structural solution the barrier analysis predicts, and the recurrent architecture in the recent periodontal literature is the same in every case: a nanoscale carrier responsible for penetration and payload protection, dispersed within a macroscale matrix responsible for placement and retention.

#### 3.7.2. Nanoparticle-in-Gel Architectures

This is the best-documented pattern. In OFI@tgel, chitosan nanoparticles carrying *Opuntia ficus-indica* polyphenols are dispersed in a hyaluronic acid/Pluronic F127 thermo-responsive gel (Section 3.3.6): the nanoparticles protect the payload from enzymatic oxidation and carry it into the biofilm, while the gel supplies residence time. The three-species eradication reported for the construct is an outcome that neither component would plausibly achieve alone [55]. In the collagenase-responsive system of Zhou and colleagues, nanoparticles of a hydrophobic PERK inhibitor are loaded into an MMP-2-cleavable hyaluronic acid hydrogel: here the nanoparticle solves dispersion of a water-insoluble drug within an aqueous network, and the matrix supplies both retention and the enzymatic trigger [62]. In the system of Zhu and colleagues, the division of labour is temporal as well as spatial: a small, freely diffusing antibiotic (minocycline hydrochloride) is released first and kills rapidly, while iron–quercetin nanoparticles released from the same ROS-cleavable network subsequently scavenge peroxide and reprogramme macrophages [64]. Sequential, staged release of this kind is the distinctive functional gain of the architecture and cannot be obtained from a single-component system.

#### 3.7.3. Other Pairings

Three further pairings appear in the literature surveyed. Nanoparticle-in-fibre systems electrospin the drug or a drug-loaded particle into a mesh that simultaneously serves as a guided-tissue-regeneration barrier and a reservoir, combining a mechanical and a pharmacological function in one device [81,82,83]. Responsive chemistry combined with an antioxidant payload, discussed in Section 3.3.6, is a pairing of trigger and cargo rather than of two carriers, and produces the self-regulating loop in which the pathological signal that opens the carrier is also the substrate consumed by the released agent [64]. Biomimetic coating combined with a conventional core, as in erythrocyte-mimicking vesicles targeting *P. gingivalis*, separates targeting (supplied by the membrane) from release kinetics (governed by the core), so that each can be optimised independently [88].

#### 3.7.4. Design Rules and the Price of Complexity

Three rules recur. First, assign each barrier to a distinct component rather than seeking a single material that addresses all of them, since the physicochemical requirements conflict—low viscosity for placement against high viscosity for retention and high cationic charge for mucoadhesion against moderate charge for EPS penetration. Second, verify that the properties of the nanocarrier survive incorporation: particle size, zeta potential and encapsulation efficiency must be characterised within the matrix and not only in suspension, since gelation can aggregate particles. Third, match the release hierarchy to the pathology, releasing the antimicrobial component early and the immunomodulatory or regenerative component over a longer horizon, as the periodontal wound-healing sequence requires [64].

The price is a compounded manufacturing and regulatory burden. A hybrid system requires two validated processes and the demonstration that the second does not compromise the first; its characterisation set is the union of both; and, for regulatory purposes, it is more likely to be classified as a combination product, with the additional complexity noted in Section 4.1.4. This is the principal reason why, despite consistent preclinical superiority, none of the hybrid periodontal systems cited here have entered clinical evaluation, and why Table 2 assigns the class a preclinical maturity. The same material, synthesised by dosage form and manufacturing route rather than by mechanism, is set out in Table 3.

**Table 3:** Synthesis of biomimetic and locally active delivery systems for the periodontal pocket. Each system class is mapped to the principal oral barrier it addresses, its key formulation determinants, release behaviour, and translational maturity, ordered by decreasing clinical validation. Representative references from the present review are indicated in the final column. The final row, phytocompound-loaded systems, is a cross-cutting payload strategy rather than a system class: it inherits the carrier of whichever class it is combined with, is placed last for that reason, and is excluded from the clinical-validation ordering that governs the preceding rows. Rows are otherwise ordered as stated. The relationship between this table, which is organised by dosage form and manufacturing route, and Table 2, which is organised by mechanism, is set out at the beginning of Section 3.

System Class	Primary Oral Barrier Addressed	Key Formulation Determinants	Release Mechanism/Kinetics	Translational Maturity & Scalability	Refs
Mucoadhesive films, chips & patches (chitosan, HPMC, carbomer)	Salivary clearance; mucosal retention	Mucoadhesive polymer; swelling/wetting; adhesion strength; film integrity	Matrix diffusion + erosion; sustained (days)	Marketed/clinical (e.g., chlorhexidine chip, PerioChip^®^); solvent casting, scalable	[19,26,41,50]
PLGA microspheres/microparticles	Pocket retention vs. clearance	Polymer MW & lactide:glycolide ratio; particle size; drug loading; degradation rate	Erosion + diffusion; sustained (weeks)	Marketed/clinical (minocycline microspheres, Arestin^®^); emulsion/spray-drying, established	[21,37,70,71]
In situ/thermosensitive gels (poloxamer, chitosan)	Salivary & crevicular clearance (residence time)	Sol–gel transition temperature; gelation kinetics; polymer concentration; viscosity	Diffusion-controlled; sustained, often with an initial burst	Early clinical; simple process, fully scalable	[49,52,53,57,58]
Polymeric nanoparticles	Biofilm matrix (EPS) penetration	Particle size (<200 nm); zeta potential (cationic → EPS interaction); surface ligands	Diffusion + matrix degradation; tunable	In vitro → animal; one negative RCT; moderate scalability	[44,69,72]
Solid lipid NPs/NLCs	Mucus & biofilm penetration; lipophilic-drug solubility	Lipid matrix composition; particle size; surfactant; encapsulation efficiency	Lipid-controlled; sustained	In vitro → animal; low clinical; high-pressure homogenisation, scalable	[73,74,75,76]
Electrospun nanofibres	Pocket topology; local depot + barrier membrane (GTR/GBR)	Fibre diameter; polymer blend; porosity; drug spatial distribution	Burst-then-sustained; programmable (multilayer/core–shell)	In vitro → preclinical; electrospinning—scale-up & sterility challenges	[81,82,83]
Hybrid nanoparticle-in-gel systems	Clearance and EPS penetration simultaneously	Nanoparticle size/charge retained after gelation; matrix trigger (thermal, enzymatic, ROS); loading of both phases	Sequential: fast small-molecule phase, then sustained nanoparticle phase	Preclinical (rodent); two validated processes required, combination-product pathway	[55,62,64]
Cell-membrane-cloaked NPs (RBC/macrophage/platelet)	Immune evasion; targeting; biofilm interfacing	Membrane source & coating integrity; core NP; membrane:core ratio	Governed by core; membrane modulates targeting, not rate	Proof-of-concept in vitro/early animal; low—batch variability, GMP hurdles	[18,85,86,88,98]
Extracellular vesicles/exosomes	Cell uptake; immunomodulation; regeneration	Source cell; isolation method; cargo-loading strategy; purity	Fusion/uptake-mediated	Early preclinical; low—yield and standardisation, GMP	[90,91,92]
ECM- & antimicrobial-peptide–mimetic systems	Regeneration + biofilm antimicrobial action	Peptide sequence/design; scaffold architecture; mechanical properties; conjugation chemistry	Entrapment/conjugation-dependent	In vitro → animal; moderate—peptide-synthesis cost	[93,94,95,96,97]
Phytocompound-loaded systems (curcumin, berberine, essential oils, polyphenols)—cross-cutting payload	Payload solubility/stability/bioavailability; AMR-sparing action	Encapsulation addressing poor aqueous solubility; loading efficiency; protection from degradation	Carrier-dependent	Mostly in vitro, some animal, one negative RCT; inherits the carrier process	[10,38,39,49,72,76,99,102]

### 3.8. Models for the Evaluation of Antibiofilm Delivery Systems

Any system is only as credible as the model on which it was tested. Part of the preclinical-to-clinical gap stems from the limitations and lack of standardisation of experimental models.

#### 3.8.1. In Vitro Biofilm Models

Monospecies models offer reproducibility and control but oversimplify the polymicrobial oral community [104]. Multispecies models reproduce the structure and resistance of the community more faithfully, at the cost of reduced reproducibility [104]. Flow systems mimic shear forces and nutrient flux better than static ones [105].

#### 3.8.2. Ex Vivo Models and Realistic Substrates

Growing the biofilm on slices of enamel, dentine, or mucosa reproduces real surface topography and chemistry [104,106]. Human or artificial saliva reproduces the acquired pellicle and fluidic conditions, increasing the relevance of retention evaluation [104].

#### 3.8.3. Evaluation of Penetration, Retention, and Release

Biofilm penetration is quantified by confocal microscopy with fluorescent markers, a more informative endpoint than the simple reduction in viability [69]. Retention and release kinetics are evaluated through detachment-force tests and profiles under salivary-flow conditions [26]. For intra-pocket systems, standardised lap-shear testing against porcine gingiva and human enamel provides a directly comparable adhesion figure and should be reported routinely [64].

#### 3.8.4. In Vivo Models

Animal models of periodontitis (rodents, ligatures or inoculation) allow evaluation in a full physiological context, but anatomical and microbiological differences limit extrapolation [107]. Their correct position is as a bridge between in vitro or ex vivo testing and clinical studies [108,109].

#### 3.8.5. The Need for Standardisation

Variability in species, growth conditions, methods, and reporting makes results difficult to compare and sometimes contradictory [104,109]. This heterogeneity explains why systems with promising preclinical results do not advance clinically [109]. A methodological consensus would increase translational value and make quantitative synthesis (meta-analysis) possible; it is this same heterogeneity that dictated the narrative rather than systematic format of the present review (Section 2.1). The phytocompound-loaded systems referred to throughout Section 3.1.7 to Section 3.6 are itemised, with their carriers, models and quantitative outcomes, in Table 4, and the qualifications that follow it illustrate directly the model heterogeneity described in this section.

**Table 4:** Representative phytocompound-loaded local delivery systems evaluated in oral biofilm and hard-tissue models. Each value is reproduced from the primary report cited in the final column and is itemised, against its source, in Supplementary Material S1; full citations are given in the reference list. Six qualifications that must be read with this table are stated immediately after it.

Phytocompound	Delivery System	Test Model	Target	Main Result	Ref.
Curcumin (polyphenol)	Mesoporous silica nanoparticles (sustained release)	In vitro (disc diffusion; release to day 45)	*P. gingivalis* (clinical isolate)	Susceptible at 6.25–50 µg/mL; largest inhibition zone at 50 µg/mL; slow release to day 45	[39]
Curcumin (polyphenol)	Poloxamer 407/Carbopol 934P in situ gel, 2%	In vitro + clinical (adult periodontitis, 1 month)	Periodontal pocket	Gelation 28–34 °C; syringeable 21 G; stable 3 months at 4 °C; significant reduction in probing depth and bleeding index vs. mechanical therapy alone	[49]
Curcumin (polyphenol)	PLGA/PLA nanoparticles, 50 µg, single application	RCT, split-mouth, double-blind, 20 patients, 180 days	Periodontal pocket	No additive benefit over SRP + empty nanoparticles for probing depth, attachment level or bleeding; transient IL-6 reduction at day 3 only—negative trial	[72]
EGCG (green-tea catechin)	Chitosan nanoparticles (ionic gelation, ~500 nm)	In vitro biofilm (67 h)	*S. mutans*	Altered cell morphology and biofilm architecture; disassembled the biofilm matrix without reducing bacterial viability, dry weight or acidogenesis	[45]
Berberine (alkaloid)	Nanoparticles (wet-milling, 40–65 nm)	In vitro	*S. mutans*	MIC/MBC 78.1/312.5 µg/mL; inhibited biofilm formation; FE-SEM cell-surface damage; improved dispersibility/bioavailability	[38]
Cinnamaldehyde (aldehyde)	Chitosan nanocapsules	In vitro + in vivo (rat caries)	*S. mutans*	Antibacterial/antibiofilm; downregulated QS, virulence and biofilm genes; reduced caries, low toxicity	[44]
Clove essential oil (*Eugenia caryophyllus*), eugenol-rich	Nanoemulsion (117 nm)	In vitro + ex vivo (bovine incisor)	*S. mutans*	MIC 0.78 mg/mL (780 µg/mL), MBC 1.56 mg/mL (1560 µg/mL); ~68% reduction in pre-formed biofilm biomass at 3.12 mg/mL (3120 µg/mL); safe in genotoxicity/cytotoxicity assays	[40]
Quercetin (flavonoid)	Hollow mesoporous silica nanocomposites (Q@HMSNs)	In vitro (erosion/abrasion; dentine specimens)	Dentine (MMP inhibition, tubule occlusion)	Promoted dentinal tubule occlusion and preserved the demineralised organic matrix, inhibiting erosion/abrasion; biocompatible with gingival fibroblasts and pulp stem cells	[80]
Quercetin (flavonoid)	Fe–quercetin nanoparticles (~2 nm) in ROS-responsive HA-PBA/PVA hydrogel, co-loaded with minocycline	In vitro + in vivo (rat ligature/*P. gingivalis* periodontitis)	*P. gingivalis*; hPDLSCs; macrophages; alveolar bone	ROS-triggered release; 77.1% DPPH scavenging; M1→M2 polarisation via Nrf2/NF-κB; protected hPDLSCs and restored osteogenesis under oxidative stress; reduced alveolar bone loss	[64]
*Opuntia ficus-indica* extract (polyphenol-rich)	Microfluidic chitosan nanoparticles in HA/Pluronic F127 thermo-responsive gel (OFI@tgel)	In vitro biofilm (36 h) + macrophage assays	*S. mutans, P. aeruginosa* (PAO1), *P. gingivalis*	Eradicated all three biofilms over 36 h; disrupted EPS formation; inhibited M1 and promoted M2 macrophage polarisation	[55]
*Zataria multiflora* aqueous extract (thymol/carvacrol-rich)	Nanoliposomes (Z-NLP, ~79 nm)	In vitro (+ enamel specimens)	*S. mutans*	Antibacterial, antibiofilm and anti-attachment; inhibited attachment to enamel comparably to 0.2% chlorhexidine	[76]

Potency values are reproduced in the units used by the source and are additionally expressed in µg/mL in the “Main result” column so that rows can be compared directly; readers should nevertheless note that the underlying assays differ in exposure time, inoculum and medium, and that these were not standardised across the primary reports. Where the source reported no free-compound or vehicle comparator, none is given here, and the absence is itself informative. The extraction sheet underlying this table, in which every value is listed against the source from which it was taken, is provided as Supplementary Material S1.

## 4. Translational Status and Future Directions

This section interprets the synthesised evidence. It first situates each formulation class along the laboratory-to-clinic gradient and then identifies the principal research gaps and future directions that condition clinical translation.

### 4.1. Translational Status: From the Laboratory to the Clinic

A pharmaceutical-technology review must confront the practical question: what has actually reached the patient? We distinguish between what is clinically available, what is in development, and what remains a preclinical concept. These three tiers, together with the barrier addressed, the key formulation determinants, and the release behaviour of each system class, are consolidated in Table 3 (Section 3.7).

#### 4.1.1. Systems Already in Clinical Use: Marketed Products, Regulatory Status and Clinical Outcomes

Intra-pocket sustained-release delivery systems—fibres, gels and microspheres with chlorhexidine, doxycycline or minocycline—have been approved as adjuncts to mechanical debridement, clinically validating the principle of local delivery [13]. The specific products, their regulatory status and their clinical outcome data are set out below, since without them the assertion of clinical validation cannot be assessed.

Chlorhexidine chip (PerioChip®): Dexcel Pharma Ltd., Or Akiva, Israel^®^; 2.5 mg chlorhexidine gluconate in a biodegradable cross-linked hydrolysed gelatin matrix; approved by the US Food and Drug Administration in 1998 as an adjunct to SRP in pockets ≥ 5 mm). The pivotal evidence comes from two double-blind, randomised, placebo-controlled multicentre trials pooled across ten centres and 447 patients. At nine months, mean probing depth reduction was 0.95 ± 0.05 mm for chip plus SRP against 0.65 ± 0.05 mm for SRP alone and 0.69 ± 0.05 mm for placebo chip plus SRP (*p* < 0.001 for both comparisons); clinical attachment level gain was 0.75 ± 0.06 mm against 0.58 ± 0.06 mm and 0.55 ± 0.06 mm respectively (*p* < 0.05). The proportion of patients achieving a probing depth reduction of at least 2 mm, the threshold usually regarded as clinically meaningful, was 19% with the chip against 8% with SRP alone (*p* < 0.05). Adverse effects were minor and transient, predominantly toothache [19].

Doxycycline hyclate gel (Atridox^®^): Tolmar Inc., Fort Collins, CO, USA; 50 mg doxycycline hyclate, equivalent to 42.5 mg doxycycline, giving a constituted concentration of 10% doxycycline hyclate, in a flowable 36.7% poly(DL-lactide)/63.3% N-methyl-2-pyrrolidone polymer that solidifies on contact with crevicular fluid and releases over seven days; FDA-approved 1998). Two multicentre studies, each enrolling 411 patients, compared the gel used as monotherapy against SRP alone, placebo vehicle and oral hygiene alone; at nine months the improvements in probing depth and attachment level obtained with the gel alone were equivalent to those obtained with SRP alone [20]. The clinical position of this product is therefore that of an alternative to instrumentation in selected sites rather than an additive adjunct, a distinction frequently lost in summary statements.

Minocycline microspheres (Arestin^®^): OraPharma, Inc., Bridgewater, NJ, USA. 1 mg minocycline hydrochloride in bioresorbable poly(glycolide-co-DL-lactide) microspheres, 20–60 µm, self-retaining and bioadhesive within the pocket; FDA-approved 2001). In two concurrent multicentre trials randomising 748 patients in total with moderate to advanced periodontitis to SRP alone, SRP plus vehicle, or SRP plus microspheres, with re-application at three and six months, the microsphere arm showed significantly greater probing depth reduction than SRP alone from the first month onwards and throughout the nine-month trial. The absolute magnitude of the additional benefit was, however, modest: mean probing depth fell by approximately 1 mm in all three arms, with the microsphere arm exceeding SRP alone by approximately 0.3 mm [21].

Two qualifications follow from these figures and are important for the argument of this review. First, the incremental effect of the marketed adjunctive antimicrobials is of the order of 0.3 mm of probing depth, close to the 0.5–0.8 mm measurement error of periodontal probing, which is why the proportion of sites achieving a clinically meaningful ≥2 mm change is a more informative endpoint than the group mean [19,21]. Second, the antibiotic-containing products carry a measurable ecological cost: minocycline microspheres transiently raised the proportion of minocycline-resistant commensal isolates in saliva and subgingival plaque, as set out in Section 3.6.5 [37]. Modest efficacy combined with demonstrable, if transient, resistance selection is precisely the gap that the phytocompound-loaded and biomimetic systems reviewed here aim to fill, and it defines the benchmark any new system must exceed. That these products exist at all shows that the translational barrier is not one of principle [14]; Section 4.1.5 considers what it is instead.

#### 4.1.2. Systems in Clinical and Advanced Preclinical Development

Nanoparticulate and mucoadhesive formulations with phytocompounds are largely at this level, with consistent in vitro/ex vivo evidence and limited clinical studies [17]. The principal obstacle is the transition from small-scale efficacy to randomised trials of adequate size [14]. The one randomised, placebo-controlled, double-blind split-mouth trial of a nanoencapsulated phytocompound in the periodontal pocket is instructive and, importantly, negative: twenty patients received SRP plus PLGA/PLA nanoparticles loaded with 50 µg curcumin or SRP plus empty nanoparticles, and probing depth, attachment level and bleeding on probing improved similarly and significantly in both arms over 180 days, with no difference in gingival crevicular fluid IL-1α, IL-6, TNF-α or IL-10 other than a transient IL-6 reduction at three days in the curcumin arm; the authors concluded that a single local application of nanoencapsulated curcumin conferred no additive benefit [72]. Set against the positive one-month clinical result obtained with a repeatedly applied 2% curcumin in situ gel [49], the contrast suggests that dosing schedule and residence time, rather than the payload itself, may determine clinical outcome—a hypothesis that the field has not yet tested directly and that we identify as a priority in Section 4.2.2.

#### 4.1.3. Systems at the Preclinical-Concept Level

Cloaked carriers, exosomes, peptide mimetics, and complex responsive systems have remarkable laboratory results but very few or no clinical studies [98]. The gap reflects the complexity of manufacturing and characterisation (Section 3.5 and Section 3.8). The hybrid architectures of Section 3.7 belong to this tier as well: the periodontal nanoparticle-in-gel systems with the most complete preclinical datasets [55,62,64] have all stopped at the rodent model.

#### 4.1.4. Regulatory and Manufacturing Barriers

Reproducibility at industrial scale is difficult for complex nanoparticulate systems [84], and more so for biologically derived ones, whose isolation yield and characterisation are not yet standardised [90]. The regulatory framework for combination products is more complex and less clear [90]. To this is added the assessment of long-term safety, relevant for metallic nanoparticles [79]. For hybrid systems the burden compounds, since two manufacturing processes must be validated and the stability of the nanocarrier within the matrix demonstrated over shelf life (Section 3.7.4).

Three requirements that are routinely omitted from formulation reports belong here, since for an intra-pocket product they are not minor. First, sterility: a device placed into an inflamed, bleeding periodontal pocket must be supplied sterile or at a defined and justified bioburden, and terminal sterilisation is problematic for most of the systems reviewed—gamma irradiation degrades polyphenols and cleaves the ROS-sensitive and enzyme-sensitive linkages on which responsive systems depend, heat destroys thermosensitive gels and lipid particles, and ethylene oxide leaves residues in porous matrices—so that aseptic processing, with its associated cost and validation burden, is usually the only route. Second, preservation and shelf-life stability: the marketed products are stable, dry or semi-solid systems, whereas aqueous nanoparticle dispersions and hydrogels are prone to aggregation, Ostwald ripening, payload oxidation and matrix hydrolysis on storage, and the only intra-pocket phytocompound gel with published stability data was demonstrated over three months at 4 °C [49], which is a research and not a commercial shelf life. Third, regulatory classification: an intra-pocket insert that acts through a released drug but is presented as a solid device is a drug–device combination product, and its route depends on the determination of the primary mode of action, which fixes the lead review division, the evidentiary standard and the timeline; hybrid nano-in-macro systems and antimicrobial-peptide-functionalised scaffolds, in which material and pharmacological functions are deliberately inseparable, are the hardest cases. None of these three is a scientific obstacle, but each adds cost and time, and their omission from the preclinical literature is one reason why estimates of translational readiness in this field are optimistic.

#### 4.1.5. Synthesis of the Maturity Gradient

The three tiers describe a gradient: clinical efficacy decreases and innovation increases from the classical systems toward the biomimetic and responsive ones. The translational message is that progress depends less on new mechanisms and more on the consolidation of existing ones through standardisation, adequate clinical studies, and the resolution of manufacturing and regulatory barriers. The quantified benchmark set by the marketed products in Section 4.1.1, roughly 0.3 mm of additional probing depth reduction, with transient resistance selection, gives that consolidation a concrete target.

A competing explanation of the same observation should be considered, because it leads to different recommendations and because the evidence favours it at least as strongly. The account above locates the bottleneck in manufacturing complexity, standardisation and regulatory pathways. Against this stands the fact that the “established” tier has not expanded in more than two decades even though its members—a cross-linked gelatin chip, a polymer gel, and PLGA microspheres—are technically straightforward to manufacture at scale, whereas complexity-driven stagnation ought to spare precisely those systems. The alternative explanation is economic and follows from the effect size. Meta-analysis of adjunctive locally delivered antimicrobials in periodontitis therapy places their benefit over subgingival instrumentation alone at a few tenths of a millimetre of additional probing depth reduction with limited certainty of evidence, and the current European clinical practice guideline accordingly gives them only a conditional recommendation for specific residual sites rather than for routine use [110,111]. If the incumbent benchmark is 0.3 mm and the clinical headroom above instrumentation is small, then a trial powered to demonstrate the superiority of a new system over an existing adjunct must be large, long and multicentre, and the commercial return on a site-applied adjunct in a price-sensitive dental market does not support that investment. On this reading the barrier facing a cell-membrane-cloaked nanoparticle is not principally that it is difficult to manufacture under good manufacturing practice, but that the trial required to prove it worth using cannot be financed by the indication. The two explanations are not exclusive, and we do not claim to adjudicate between them, but the second has three practical implications that the first does not. Development should target the populations in which the headroom is largest—deep residual pockets, furcations, non-responding sites, smokers and patients with diabetes—rather than the average site. Endpoints should be chosen where a new mechanism could plausibly exceed 0.3 mm, such as the proportion of sites reaching pocket closure, the avoidance of surgery, or ecological and inflammatory outcomes, rather than mean probing depth change. And systems whose additional function is regenerative or immunomodulatory rather than antimicrobial should be evaluated against the endpoints proper to that function, since it is there, and not in incremental antimicrobial efficacy, that the available headroom lies.

### 4.2. Research Gaps and Future Directions

#### 4.2.1. Methodological Standardisation of Evaluation

The most immediately solvable gap is the lack of common protocols (Section 3.8). Representative multispecies models, realistic flow conditions, and a minimum set of endpoints, including penetration and retention, would make results comparable and would permit quantitative synthesis [104,109]. A specific and tractable addition, identified in Section 3.6.5, is the systematic performance of serial-passage resistance-induction experiments with oral pathogens (*S. mutans*, *P. gingivalis*, *A. actinomycetemcomitans*) under sub-inhibitory phytocompound pressure, in parallel with the antibiotics used in local therapy; such data exist for staphylococci and enterobacteria [102] but not for the oral community, and their absence is the weakest link in the resistance-sparing argument.

#### 4.2.2. Randomised Controlled Trials of Adequate Size

Randomised controlled trials with adequate samples, standardised endpoints, and sufficient follow-up are needed to confirm whether preclinical efficacy translates clinically [14]. The design of such trials should be informed by the discrepancy noted in Section 4.1.2: single-application nanoparticle delivery of curcumin failed to add benefit to SRP [72], whereas repeated application of a retentive curcumin gel did [49]. Dosing schedule and residence time, not payload identity, should therefore be treated as primary design variables, and trials should be powered on the proportion of sites achieving ≥2 mm probing depth reduction rather than on group mean change, given the 0.5–0.8 mm measurement error of probing [19].

#### 4.2.3. Integration with the Microbiome and Precision Medicine

One prospective direction is the shift from biofilm destruction to modulation of the oral ecosystem, selectively targeting pathogens while protecting the commensal community [112]. Coupling with individual microbiome profiling would open the way to personalised delivery, a natural bridge toward pharmacomicrobiomics [3,112,113,114]. The transient but measurable selection of resistant commensals after local antibiotic delivery [37] is a concrete argument for making ecological endpoints, and not only clinical indices, part of the routine assessment of any new local system.

#### 4.2.4. Positioning Against Competing and Complementary Local Adjuncts

Local pharmacological delivery is not the only adjunct competing for the same clinical space, and a review that omits the alternatives overstates its subject. Three are directly relevant. Antimicrobial photodynamic therapy delivers a photosensitiser subgingivally and activates it with light. A systematic review found no statistically significant additional probing depth reduction over instrumentation alone in the pooled analysis (weighted mean difference 0.35 mm, 95% CI −0.04 to 0.73), with the evidence limited by few controlled studies and heterogeneous designs [115]. Host-modulation therapy addresses the destructive host response rather than the biofilm, and subantimicrobial-dose doxycycline, which inhibits matrix metalloproteinases at doses below those exerting antibiotic selection pressure, is its established example and the closest existing analogue to the dual-function systems advocated in Section 4.2.5 [116,117]. Probiotics and live biotherapeutics pursue ecological modulation rather than eradication, which is the strategy argued for in Section 4.2.3, and their adjunctive effects on clinical parameters remain small, strain- and regimen-dependent, and too heterogeneous to support firm recommendations [118]. Each of these competes for the same narrow band of clinical headroom above instrumentation (of the order of 0.3 mm) identified in Section 4.1.5, and a new delivery system must be positioned against them and not only against instrumentation alone.

The incumbent within local delivery also deserves a fairer accounting than it usually receives. Chlorhexidine is effective and inexpensive, but its liabilities are real: extrinsic staining of teeth and restorations, taste disturbance, mucosal desquamation, dose-dependent cytotoxicity to gingival fibroblasts and periodontal ligament cells at concentrations well below those used clinically, and the reduced susceptibility and cross-adaptation discussed in Section 3.6.5 [103]. Presenting the “established” tier without these qualifications flatters it, and it also understates the opportunity: a locally delivered agent that matched chlorhexidine’s efficacy without staining, without fibroblast toxicity and without selecting for reduced susceptibility would be clinically valuable even if it produced no additional probing depth reduction at all. We suggest that this, rather than incremental millimetres, is the most realistic near-term target for the phytocompound-loaded and biomimetic systems reviewed here.

#### 4.2.5. Multifunctional and Dual-Function Systems: Definition and Examples

The concept is defined here explicitly. A conventional delivery system is pharmacologically inert: its carrier serves only to place, protect and release the active agent, and if the carrier were replaced by an equally retentive but chemically different vehicle, the therapeutic outcome would, in principle, be unchanged. A multifunctional or dual-function system is one in which the carrier itself makes a measurable therapeutic contribution that is separable from, and additional to, the release of its payload, so that the drug-free carrier is not therapeutically equivalent to no treatment. The operational test proposed here, and satisfied by the studies cited below, is that the unloaded carrier shows a significant effect against a relevant biological endpoint in its own control arm.

Three distinct forms of this dual function appear in the oral literature. In the first, the carrier is intrinsically antimicrobial: chitosan’s protonated amino groups destabilise the bacterial envelope independently of any encapsulated drug, so a chitosan nanoparticle is simultaneously vehicle and antibacterial agent (Section 3.2.2) [42]. In the second, the carrier is chemically consumed by the pathological signal that triggers it: in the ROS-responsive borate-ester networks of Section 3.3.5, oxidation of the linker both releases the payload and removes peroxide from the periodontal microenvironment, so that scavenging capacity is a property of the matrix and not only of the cargo; in the study of Zhu and colleagues, the drug-free hydrogel arms allow this contribution to be separated experimentally from that of the released minocycline and quercetin [64]. In the third, the carrier is regenerative: collagen and hyaluronic acid matrices provide a substrate for cell attachment and remodelling and, in the case of hyaluronic acid, modulate healing and inflammation directly, so that the scaffold contributes to tissue repair while releasing its agent [46,93,94]. Mineralised scaffolds bearing covalently anchored antimicrobial peptides combine the second and third forms, since the antimicrobial function resides in the material surface rather than in a diffusible payload [94].

Two further points follow. Phytocompound loading is particularly well matched to this design, because the antioxidant payload and a ROS-consuming matrix act on the same pathological variable through independent routes, producing the self-regulating loop described in Section 3.3.6 [64,68]. And the design imposes a specific evaluation requirement that is often neglected: because the carrier is therapeutically active, the appropriate control is the drug-free carrier rather than an untreated site, and studies that omit this arm cannot attribute their effect between carrier and payload. Representative phytocompound-loaded systems evaluated in oral biofilm and hard-tissue models are summarised in Table 4 (Section 3.8).

#### 4.2.6. Resolving Manufacturing and Regulatory Barriers

Scalable production methods, characterisation standards for biologically derived systems, and clearer regulatory pathways are the conditions for advancing complex systems [15,84]. Without these advances, the most elegant mechanisms remain in the laboratory.

Six qualifications must be read with this table, and they are stated here rather than left to the reader to infer. (i) Units and comparators. Potencies span three orders of magnitude and are reported in mixed units by their sources: on a common basis the values are approximately 6.25–50 µg/mL for curcumin–silica against *P. gingivalis* [39], 78.1 µg/mL for berberine nanoparticles against *S. mutans* [38], and 780 µg/mL for the clove-oil nanoemulsion against the same organism [40]. Only the *Zataria* row carries a comparator arm (0.2% chlorhexidine) [76]; elsewhere the free-compound minimum inhibitory concentration is not reported alongside the formulated one, so the contribution of encapsulation cannot be separated from the intrinsic activity of the molecule, and no row in this table demonstrates that nanoformulation improved potency. (ii) Exposure time. Where the source specified it, it is given in the “Test model” column (36 h, 67 h, 45 days); several sources did not specify it, and comparison across rows is correspondingly weak. (iii) Model. Most entries are monospecies assays in static in vitro systems, which Section 3.8.1 identifies as an oversimplification of the polymicrobial oral community. We therefore do not treat this table as evidence of clinical promise; it is a map of what has been measured, and the discount that Section 3.8.1 applies to monospecies static data applies to it in full. Five entries carry correspondingly more weight and are identified here: the *Opuntia* system was tested against three species [55]; the cinnamaldehyde and quercetin entries include in vivo rodent data [44,64]; the curcumin in situ gel was assessed clinically [49]; and the curcumin nanoparticle entry is a randomised controlled trial, which was negative [72]. (iv) The EGCG entry is a negative result on the endpoints that matter cariologically and is retained deliberately rather than tacitly. Disassembly of the biofilm matrix without any reduction in bacterial viability, dry weight or acidogenesis [45] means the formulation altered biofilm architecture without reducing the acid challenge to enamel; it is evidence of an anti-matrix mechanism and evidence against a cariostatic effect, and it is a useful illustration of why matrix-directed endpoints must not be reported as antimicrobial ones. (v) The quercetin–silica entry [80] concerns dentine erosion, abrasion and tubule occlusion and has no antibiofilm content; it is retained because quercetin recurs as a payload elsewhere in this review, but it carries no weight in the antibiofilm argument and should not be read as doing so. (vi) The berberine particle size of 40–65 nm was re-checked against the source and is as reported there [38]; it is unusually small for top-down wet media milling, which more commonly yields 100–500 nm, and the source attributes it to the milling conditions and stabiliser system used. We flag it because it is an outlier for the process rather than because we doubt the report, and independent confirmation would be valuable.

## 5. Conclusions

Maintaining and restoring oral health in biofilm-mediated diseases remains limited by a fundamental pharmaceutical obstacle: the difficulty of reaching and maintaining therapeutic concentrations at the site, against salivary and crevicular clearance, the mucosal barrier, and the protective biofilm matrix. The systems analysed each address part of this obstacle: mucoadhesives provide retention, in situ gels reconcile application with localisation, particulate systems penetrate the biofilm, and biomimetic systems add intrinsic therapeutic functions and mechanisms with a reduced risk of resistance.

The common thread is that no isolated system resolves all barriers simultaneously, and that maximum value results from the rational integration of several strategies. The hybrid nanoparticle-in-gel architectures analysed in Section 3.7 are the concrete expression of this conclusion: a nanocarrier supplies penetration and payload protection, a responsive or thermosensitive matrix supplies placement, retention and the release trigger, and the two together achieve staged release that neither component achieves alone—at the cost of a compounded manufacturing and regulatory burden that has so far kept every such system at the rodent stage. Loading these architectures with bioactive phytocompounds is particularly promising, since their simultaneous antioxidant, anti-inflammatory, and antimicrobial profile aligns with the multifactorial nature of oral disease and offers a route to reducing dependence on antibiotics and the selection pressure for resistance; the resistance-sparing argument rests, however, on a mechanistic rationale and on in vitro serial-passage data obtained in non-oral species, and awaits direct testing in the oral community.

At the same time, this review has honestly emphasised the persistent gap between the richness of mechanisms demonstrated preclinically and the rarity of clinical validation. The marketed products define the benchmark quantitatively: approximately 0.3 mm of additional probing depth reduction over SRP alone at nine months, with a transient increase in resistant oral isolates for the antibiotic-containing formulations. Progress today depends less on the invention of new mechanisms and more on the consolidation of existing ones: standardisation of methodology, adequate randomised clinical trials, integration with an understanding of the microbiome, and the resolution of manufacturing and regulatory barriers. The most fertile direction appears to be the convergence of formulation technology, the redox pathophysiology of the oral cavity, and the strategy of combating antimicrobial resistance, a convergence in which local delivery systems for phytocompounds occupy a central position. Two constraints identified quantitatively in this review should govern that convergence. The pocket arithmetic of Section 3.1.7 shows that, for payloads whose minimum inhibitory concentrations lie in the mg/mL range, deliverable mass rather than carrier retention is the limiting factor, which argues for potentiation, for repeated application, or for repositioning such payloads as anti-virulence rather than bactericidal agents. And the pH data of Section 3.1.4 show that the inflamed periodontal pocket is neutral-to-alkaline rather than acidic, which excludes acid-triggered release from periodontal design and identifies alkaline-triggered release as an open and rational alternative. Both are testable propositions rather than aspirations, and we offer them as the most concrete contribution of this review.

## Data Availability

No new primary data were generated. All supporting material, as itemised under Supplementary Materials above, is provided as Supplementary Material S1.

## Supplementary Materials

The following supporting information can be downloaded at https://www.mdpi.com/article/10.3390/pharmaceutics18081011/s1, Supplementary Material S1, the structured data-extraction matrix described in Section 2.5, giving for every source the system class, carrier composition, payload, oral barrier addressed, release mechanism, experimental model, principal quantitative outcome and level of evidence, together with an itemised list of the values reproduced in Table 1 and Table 4 and the source of each, and the full search strings of Section 2.2 and the record counts underlying Figure 1.

## Abbreviations

ABTS, 2,2′-azino-bis(3-ethylbenzothiazoline-6-sulfonic acid); AMP, antimicrobial peptide; AMR, antimicrobial resistance; Arg-1, arginase-1; CAL, clinical attachment level; CAT, catalase; DPPH, 2,2-diphenyl-1-picrylhydrazyl; ECM, extracellular matrix; EGCG, epigallocatechin-3-gallate; EPS, extracellular polymeric substance; FDA, US Food and Drug Administration; FE-SEM, field-emission scanning electron microscopy; GBR, guided bone regeneration; GCF, gingival crevicular fluid; GMP, good manufacturing practice; GTR, guided tissue regeneration; HA, hyaluronic acid; hPDLSC, human periodontal ligament stem cell; HPMC, hydroxypropyl methylcellulose; IL, interleukin; MBC, minimum bactericidal concentration; MIC, minimum inhibitory concentration; MMP, matrix metalloproteinase; NF-κB, nuclear factor kappa B; NLC, nanostructured lipid carrier; NP, nanoparticle; Nrf2, nuclear factor erythroid 2-related factor 2; PBA, phenylboronic acid; PD, probing depth; PERK, protein kinase R-like endoplasmic reticulum kinase; PLGA, poly(lactic-co-glycolic acid); PRISMA, Preferred Reporting Items for Systematic Reviews and Meta-Analyses; PVA, poly(vinyl alcohol); QS, quorum sensing; RBC, red blood cell; RCT, randomised controlled trial; ROS, reactive oxygen species; SLN, solid lipid nanoparticle; SOD-1, superoxide dismutase 1; SRP, scaling and root planing; TNF-α, tumour necrosis factor alpha.
